# Advances in Molecularly Imprinted Polymers for Bone Biomarker Detection and Therapeutic Applications

**DOI:** 10.1002/open.202500127

**Published:** 2025-07-17

**Authors:** Ren Yang, Xiaohan Ma, Mingcheng Xuan, Yingqi Ma, Jiexian Ding, David Y. S. Chau, Jonathan C. Knowles, Feng Peng, Alessandro Poma

**Affiliations:** ^1^ Division of Biomaterials and Tissue Engineering Eastman Dental Institute University College London Royal Free Hospital Rowland Hill Street London NW3 2PF UK; ^2^ Department of Orthopedics, Guangdong Provincial People's Hospital (Guangdong Academy of Medical Sciences) Southern Medical University Guangzhou 510080 China; ^3^ Kavli Institute for Nanoscience Discovery University of Oxford Dorothy Crowfoot Hodgkin Building South Parks Rd Oxford OX1 3QU UK; ^4^ The Pediatric Orthopaedics The Second Hospital of Lanzhou University Lanzhou 730030 China; ^5^ Department of Nanobiomedical Science and BK21 PLUS NBM Global Research Center for Regenerative Medicine Dankook University Cheonan 31116 South Korea; ^6^ UCL Eastman‐Korea Dental Medicine Innovation Centre Dankook University Cheonan 31116 South Korea

**Keywords:** bone biomarkers, controlled drug release, molecularly imprinted polymers, osteogenic treatment, tissue engineering

## Abstract

This review explores the application of molecularly imprinted polymers (MIPs) in detecting bone turnover biomarkers and advancing osteogenic treatment strategies. MIPs, designed to mimic biological recognition sites, offer innovative solutions for precise molecular recognition in bone health management. Chemical methodologies for MIPs synthesis and their integration into diagnostic systems for detecting bone resorption markers are highlighted. Furthermore, MIP‐driven therapeutic advancements, including controlled drug release, cell imprinting for osteogenic differentiation, and functional scaffolds for tissue regeneration, are emphasized. This review underscores MIPs’ potential to revolutionize bone disease management and calls for further exploration into chemical designs to optimize their clinical and practical applications.

## Graphical abstract

This review highlights the emerging applications of molecularly imprinted polymers (MIPs) in bone regeneration and diagnosis. MIPs facilitate osteogenic treatments by guiding stem cell differentiation and delivering bioactive molecules and enable precise detection of bone turnover biomarkers via MIP‐based sensors, offering promising strategies for next‐generation bone repair and monitoring.

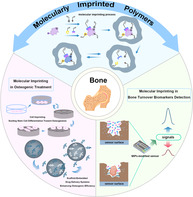

## 1 Introduction

The dynamic balance of bone tissue relies on the coordinated processes of bone resorption and bone formation, which are crucial for maintaining skeletal health and functionality^[^ ^1^ ^]^ [Florencio‐Silva, 2015 #2]. Various orthopedic diseases, such as osteoporosis,^[^ ^2^ ^]^ multiple myeloma,^[^ ^3^ ^]^ delayed fracture healing,^[^ ^4^ ^]^ and Paget's disease of bone,^[^ ^5^ ^]^ stem from an imbalance between these two processes. These conditions may lead to reduced bone density, structural deterioration, and functional impairment, significantly affecting a patients’ quality of life. Current therapeutic strategies include drug regimens (e.g., antiresorptive drugs and bone formation‐promoting drugs), surgical interventions (e.g., bone repair and implants), and physical therapies (e.g., ultrasound and electromagnetic field stimulation).^[^ ^6^, ^7^, ^8^ ^]^ Additionally, bone density measurement and the monitoring of bone resorption biomarkers have become essential tools for disease diagnosis and treatment evaluation, supporting precision therapy and personalized medicine.^[^ ^9^, ^10^ ^]^

Despite the advancements in existing treatments for orthopedic diseases, significant limitations persist, such as drug side effects, diminishing efficacy, the invasiveness of surgical procedures, and challenges in regenerating complex bone structures.^[^ ^11^ ^]^ Moreover, bone density measurements lack timeliness, and the high costs of biomarker monitoring hinder real‐time dynamic assessment, failing to fully meet clinician and patient needs.^[^ ^12^ ^]^ Advanced tissue‐engineering materials and detection technologies offer novel solutions to these challenges. Among these, molecularly imprinted polymers (MIPs) have emerged as a promising approach in orthopedic applications. MIPs are crosslinked polymer networks generated around a molecular “template,” and after template removal, they retain suitable cavities that bind the same target with antibody‐like affinity and selectivity while remaining far more robust to harsh pH, temperature, and solvents conditions.^[^ ^13^, ^14^ ^]^ Recent progress in MIPs indicates that these “synthetic antibodies” can directly overcome many of the shortcomings outlined above.

On the diagnostic front, Afsarimanesh et al. developed an interdigital capacitive sensor integrated with electrochemical impedance spectroscopy (EIS), coated with a MIPs layer selective for the C‐terminal telopeptide of type I collagen (CTX‐I), a key bone resorption marker. This sensor achieves an analytical sensitivity of 0.09 ng mL^−1^ within ≈7 min, comparable to the standard ELISA method (0.20 ng mL^−1^). However, unlike ELISA, the MIP‐based sensor eliminates the need for costly biological antibodies, significantly reduces assay time from over 3 h to just minutes, and requires only 50 μL of sample.^[^ ^15^ ^]^ These advantages collectively reduce costs and enable real‐time, point‐of‐care monitoring.

Therapeutically, Wang et al. demonstrated the translational potential of MIPs in orthopedic applications by designing pH‐responsive MIPs nanospheres templated with dexamethasone‐21‐phosphate (DXP). These MIPs nanospheres exhibited nearly twice the drug‐loading capacity of nonimprinted controls and enabled accelerated release under mildly acidic conditions (pH 6.0–7.4), which are characteristic of post‐surgical inflammation. When incorporated into a hydrogel matrix, the nanospheres facilitated controlled drug release for up to eight weeks, effectively mitigating the initial burst release typically observed with conventional hydrogels.^[^ ^16^ ^]^ This selective, stimulus‐responsive, and sustained delivery profile highlights the potential of MIPs to enhance localized anti‐inflammatory treatment and extend the operational lifespan of implantable biosensors or orthopedic devices.

Collectively, these representative studies highlight how the high affinity, stability, and stimulus‐responsive behavior of MIPs translate into faster, cheaper, and more effective diagnostic and therapeutic tools for bone‐related diseases. Consequently, MIPs offer a viable pathway to address both the cost‐performance gap in biomarker detection and the need for scaffold platforms that deliver bioactive signals with precise, stimuli‐responsive control in bone tissue engineering while also addressing the limitations of conventional antibody assays and implants material.

## 2 MIPs

### 2.1 Rationale of MIPs

**Figure 1:**
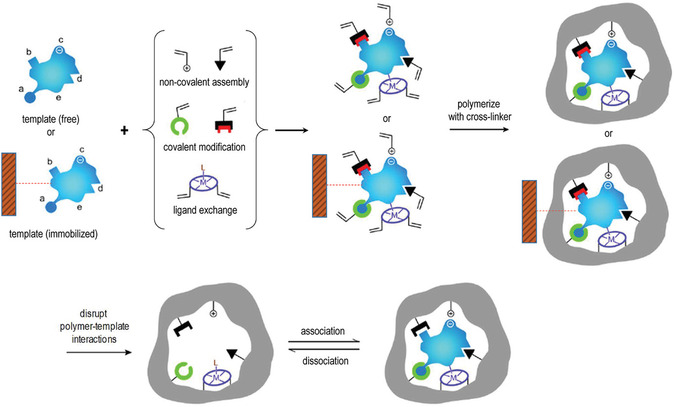
Scheme of the molecular imprinting process: the establishment of interactions between the template (free in solution or immobilized on a suitable solid support) and polymerizable groups interacting either covalently, noncovalently, or via coordination with a metal center with suitable functional groups or structural elements of the template. Subsequent polymerization in the presence of a crosslinker develops a porous insoluble matrix containing the binding sites for the template. At this point, either the template is removed (if free) or alternatively the polymer is separated from the immobilized template in suitable washing/elution conditions. In all cases, the target analyte can selectively rebind to the polymer into the sites formed by the template or “imprints.” Adapted with permission.^[^ ^18^ ^]^ Copyright, 2010, Elsevier Ltd. All rights reserved.

As briefly mentioned above, molecular imprinting enables the design of materials with high specificity and functionality, capable of mimicking biological interactions or tailoring microenvironments for therapeutic purposes.^[^ ^13^ ^]^ Specifically, MIPs are synthetic biomimetic materials designed to emulate natural antibody‐antigen systems. These materials feature specific spaces capable of recognizing and binding target molecules (“antigens”) with high affinity and selectivity.^[^ ^17^ ^]^ The formation of MIPs involves several steps: initially, functional monomers surround the template molecule, forming a template‐monomer complex through reversible covalent, noncovalent, or semicovalent interactions. This complex is then copolymerized with a crosslinking agent in an appropriate solvent. Once the template molecules are removed, recognition cavities complementary in shape, size, and chemical functionality to the template molecules are created within the crosslinked 3D polymer matrix (**Figure** **1**).^[^ ^18^ ^]^

The synthesized MIPs operate on a “lock‐and‐key” mechanism, recognizing and binding target molecules at the imprinted sites. This process closely resembles the interactions observed in receptor‐ligand, antibody‐antigen, and enzyme‐substrate systems, making MIPs versatile for a wide range of applications.^[^ ^19^ ^]^ Among the various imprinting methods, noncovalent imprinting is the most widely used due to its simplicity and efficacy in binding and removing template molecules.^[^ ^20^ ^]^

Successfully synthesized MIPs require an optimal balance of rigidity and flexibility.^[^ ^21^ ^]^ They must retain the original shape and size of the cavities after template removal while allowing for rapid substrate binding to achieve equilibrium. Compared to natural antibodies (proteins, nucleic acids, and other biological systems), MIPs offer superior physical robustness, mechanical strength, resistance to extreme temperatures and pressures, and chemical inertness toward acids, bases, metal ions, and organic solvents.^[^ ^22^ ^]^ Furthermore, MIPs are cost‐effective to produce and can be stored indefinitely under standard conditions without requiring specialized environments. Their stability across a wide temperature range makes them highly adaptable for diverse applications.^[^ ^23^ ^]^

These attributes endow MIPs with several distinct advantages over biological recognition elements such as antibodies. While antibodies exhibit high specificity, their functionality is heavily dependent on conformational stability and is easily compromised under nonphysiological conditions. In contrast, MIPs maintain their recognition capacity even in harsh chemical or thermal environments, making them suitable for real‐world applications that demand operational stability.^[^ ^23^ ^]^ Additionally, antibody production involves time‐consuming and resource‐intensive biological processes, resulting in high costs and batch variability. MIPs, on the other hand, are synthesized via controllable chemical reactions that are reproducible, scalable, and far more economical.^[^ ^21^ ^]^ Notably, MIPs do not require cold‐chain storage and retain functionality after long‐term ambient storage, an essential advantage for field‐deployable diagnostics and resource‐limited clinical settings. Together, these advantages underscore the robustness, stability, and cost‐efficiency of MIPs as next‐generation alternatives to traditional biological recognition systems.

### 2.2 Production of MIPs

The synthesis of MIPs is a highly challenging and complex chemical endeavor, as it involves numerous interdependent experimental variables. These include the nature and quantity of templates, functional monomers, crosslinkers, porogens, initiators, preparation methods, polymerization conditions, reaction times, and elution techniques.^[^ ^24^, ^25^ ^]^

Based on the composition and structure of the functional monomers as well as the nature of the polymerization reaction, the mechanisms of polymerization can be categorized into condensation polymerization and addition polymerization. Among these, free radical polymerization (FRP), a subset of addition polymerization, is the most widely used method for synthesizing MIPs.^[^ ^26^ ^]^ In recent years, the advent and development of controlled/living radical polymerization (CRP/LRP) techniques, such as atom transfer radical polymerization (ATRP), reversible addition‐fragmentation chain transfer (RAFT), and nitroxide‐mediated polymerization (NMP), have expanded their application to MIPs synthesis.^[^ ^27^ ^]^ Compared to traditional FRP, these methods allow precise control over chain growth during polymerization, enabling fine‐tuning of polymer thickness and size. This advancement facilitates the production of multifunctional MIPs materials.^[^ ^28^ ^]^

MIPs synthesized through FRP and CRP/LRP mechanisms can be prepared using various methods. The table below outlines the principles, advantages, disadvantages, and typical applications of MIPs synthesized via different preparation techniques (**Table** **1**). These methods form the foundation of MIPs production and have been widely applied across diverse fields, including solid‐phase extraction (SPE), chromatographic stationary phases, drug delivery, biosensing, and environmental monitoring.^[^ ^29^, ^30^ ^]^ Each technique offers unique advantages in terms of scalability, selectivity, particle morphology, and template compatibility, enabling tailored MIPs designs that align with the requirements of specific analytical, biomedical, or environmental applications.

**Table 1:** Common preparation method of MIPs with their advantage and limitations.

Preparation method	Principle	Advantages	Disadvantages	Typical applications	References
Bulk polymerization	Radical polymerization involves mixing templates and functional monomers for prepolymerization, followed by the addition of a crosslinker, an initiator, and a porogen. After polymerization, the resulting solid polymer is mechanically ground and sieved to obtain particles of suitable size. Finally, the polymer is eluted with a solvent to remove the template, completing the process.	Simple; convenient operation.	Time‐consuming and labor‐intensive; requires mechanical grinding and sieving, which may damage polymer integrity and recognition sites; deeply embedded imprinting sites hinder template removal and rebinding, leading to low binding capacity, slow mass transfer, and long equilibrium times.	Preparation of porous polymeric membranes with molecular recognition capabilities. For example, cyclodextrin‐based membranes prepared by bulk polymerization exhibited significantly enhanced specific surface area, enabling efficient and selective separation of environmental pollutants such as bisphenol A from aqueous solutions.	[183, 184, 185, 186]
Precipitation polymerization	The process begins by dissolving the functional monomer, template, initiator, and crosslinker in an organic solvent. Polymerization occurs within the solvent, resulting in the production of an insoluble polymer that precipitates out of the solution.	No need for surfactant and other solvent; the binding site avoid to be damaged; easier to control the particle size.	Requires large volumes of solvent and template; imprinting sites may still be embedded; limiting binding capacity.	Used in the preparation of monodisperse MIPs microspheres for SPE or high‐performance liquid chromatography (HPLC) stationary phase, or drug delivery microsystems. For example, preparation of monodisperse MIPs microspheres (≈3 μm) via precipitation polymerization for the selective extraction of matrine and oxymatrine from *Sophora flavescens* root, demonstrated higher selectivity and cleaner HPLC chromatograms than conventional C18 cartridges.	[183, 184, 187, 188, 189]
Early‐termination polymerization	Polymerization is initiated at high concentrations of functional monomers and templates but intentionally terminated early by dilution or UV‐controlled “start‐stop” methods, forming monodisperse nanoparticles or nanogels rather than bulk polymers.	High‐yield production; uniform particle size and monodispersity; effective preservation of recognition sites due to avoidance of grinding.	Initial high concentrations of monomers and templates lead to higher costs and waste; substantial solvent usage required due to dilution steps.	Preparation of nanoscale MIPs with uniform particle size and morphology, suitable for diverse applications including drug delivery, biosensing, selective separation, and enzyme‐like catalysis. For example, preparation of soluble, nanoscale enzyme‐mimicking catalysts through early‐termination polymerization, exemplified by carboxypeptidase A‐imprinted nanoparticles exhibiting superior catalytic performance in carbonate hydrolysis reactions.	[190, 191]
Emulsion polymerization	The process involves placing the monomer, template, one or more surfactants, and initiator in either the water or oil phase, followed by stabilizing the monomer droplets within the continuous phase (typically water) using surfactants. A stable emulsion is achieved through vigorous stirring or sonication, effectively dispersing the oil and water phases.	Simple operation; suitable for large‐scale synthesis; smaller particle sizes and narrow size distribution; minimal mechanical damage to binding sites.	Surfactants may interfere with molecular recognition and contaminate the final product; complex purification steps; risk of template leakage.	Preparation of uniform MIPs nanoparticles for targeted drug delivery, biomedical sensors, bioimaging probes, and chromatographic separation requiring controlled size and narrow particle size distribution. For example, preparation of paclitaxel‐imprinted nanoparticles via emulsion polymerization modified with PEG‐folic acid demonstrated high drug‐loading efficiency, pH‐responsive controlled release, and significantly enhanced uptake by cancer cells expressing folate receptors.	[192, 193, 194]
Core‐shell polymerization	Solid particles are initially formed as cores using various materials (e.g., silica, MNPs, quantum dots). Subsequently, the cores are coated with a thin shell composed of the template molecule, functional monomers, and crosslinkers. After polymerization, template removal yields recognition sites primarily located on the particle surfaces.	Easy removal of templates due to surface‐bound imprinting sites; controlled and diverse core materials; improved accessibility of binding sites.	Potentially larger particle size; limited binding capacity due to thinner shell layers; complexity in controlling shell uniformity and thickness.	Fabrication of core‐shell MIPs nanoparticles for biosensors, selective extraction, and purification (magnetic separation), fluorescence imaging (quantum dot‐based sensors), and targeted drug delivery systems, benefiting from easily accessible surface recognition sites. For example, a electrochemiluminescence (ECL) immunosensor was constructed using magnetic core‐shell MIPs as capture probes for the ultrasensitive detection of anti‐HIV‐1 antibodies, provided high enrichment capacity, reusability, and significantly improved the cost‐efficiency and stability of the immunosensor.	[195, 196, 197]
Solid‐phase polymerization	The process begins with the preparation of template‐grafting solid phase (e.g., glass beads), followed by polymerization initiated by incubating a mixture of the functional monomer, crosslinker, initiator, and the template‐bearing material. Imprinted polymers form around the template molecules. Initial washing under mild conditions, such as low temperatures, removes unreacted monomers and low‐affinity MIPs, while more drastic elution conditions, such as high temperatures, are used to elute high‐affinity MIPs from the template.	Reusable templates; relatively uniform particle size; enrichment of high‐affinity MIPs.	Time‐consuming; labor‐intensive when performed manually; limited scalability without automation.	Preparation of uniform, high‐affinity MIPs for biosensing, selective protein/peptide enrichment, and affinity purification. Particularly suited for applications requiring automation or high‐specificity recognition in complex biological samples. For example, epitope‐imprinted nanoparticles synthesized via solid‐phase polymerization were developed as artificial antibodies targeting the C‐terminus of the CB1 receptor. The MIPs demonstrated high specificity and nanometre‐scale size, enabling selective recognition of G‐protein‐coupled receptor (GPCR) fragments in Western blot and biosensing applications.	[192, 198, 199, 200, 201, 202]

As summarized in Table 1, each preparation method exhibits specific structural and functional characteristics that influence its suitability for particular applications. Bulk polymerization, while simple and cost‐effective, typically produces irregularly shaped particles with deeply embedded recognition sites that are susceptible to damage during grinding. As such, it is more suitable for macroscopic MIPs formats, such as membrane‐based separation materials or stationary sorbents. Precipitation polymerization allows for the straightforward and efficient synthesis of monodisperse microspheres, making it a preferred method for high‐performance SPE and chromatographic separation. Emulsion and early‐termination polymerizations offer greater control over particle size at the nanometer scale and are particularly advantageous in applications requiring uniform, nanosized MIPs, such as targeted drug delivery systems and biosensing platforms. Core‐shell polymerization, through the incorporation of functional core materials (e.g., magnetic or fluorescent nanoparticles), enables multifunctional MIPs with enhanced signal response or separation capability. When combined with solid‐phase polymerization, this approach allows for precise surface imprinting, affording both binding‐site accessibility and customizable core functionalities. In particular, solid‐phase polymerization excels in the selective imprinting of biological macromolecules, such as peptides or proteins, due to its reusability, affinity enrichment, and potential for automation.

These method‐specific features demonstrate that no single polymerization strategy is universally optimal, and synthesis must be matched to the physicochemical nature of the template, the desired particle morphology, and the functional requirements of the application.^[^ ^31^ ^]^ To meet the increasingly specialized demands of biomedical and analytical applications, innovative MIPs synthesis strategies are being explored.

**Figure 2:**
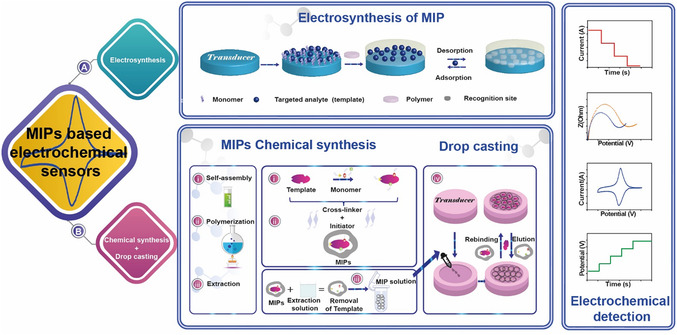
Scheme of the preparation of MIP‐based electrochemical sensors, including the electrochemical and chemical synthesis. Adapted with permission.^[^ ^181^ ^]^

Electropolymerization is a technique for synthesizing MIPs directly on conductive substrates and is particularly well‐suited for the fabrication of electrochemical sensors (**Figure** **2**). These sensors typically consist of two key components: a recognition layer, MIP layer that provides selective analyte binding, and an electrochemical transducer that converts molecular interactions into measurable signals.^[^ ^32^, ^33^, ^34^ ^]^ In this method, a template molecule first forms a noncovalent prepolymerization complex with electroactive monomers, such as pyrrole, aniline, or o‐phenylenediamine.^[^ ^35^, ^36^ ^]^ This complex is then polymerized directly onto the surface of a conductive electrode (commonly gold, carbon, or platinum) by applying a controlled electrical potential or current. The resulting redox‐initiated polymerization generates a highly crosslinked and compact conductive polymer film in which the template is embedded. After polymerization, template removal is achieved through electrochemical cleaning (e.g., cyclic voltammetric stripping) or solvent extraction, leaving behind complementary cavities capable of selective molecular recognition.^[^ ^37^, ^38^ ^]^

A key advantage of electropolymerization lies in its ability to precisely control film thickness, morphology, and surface coverage by tuning electrochemical parameters, such as applied potential, scan rate, or polymerization time.^[^ ^28^ ^]^ Furthermore, because the polymerization occurs directly on the electrode surface, no post‐deposition processing is required, facilitating sensor miniaturization and integration.^[^ ^33^ ^]^ The conductive nature of the resulting MIPs films also enhances electron transfer between the recognition layer and the transducer, improving signal sensitivity and enabling real‐time detection.^[^ ^39^ ^]^ These features make electropolymerization‐based MIPs particularly advantageous for biosensing in complex media, where high selectivity and signal fidelity are critical.^[^ ^40^ ^]^ However, this technique is limited to electroactive monomers, and the template removal process can sometimes involve harsh electrochemical or chemical conditions that risk damaging the underlying electrode surface or impairing recognition site integrity.^[^ ^41^ ^]^ Despite these challenges, electropolymerized MIPs have demonstrated excellent performance in various sensing platforms, including the ultrasensitive detection of biomarkers in blood, owing to their enhanced selectivity, stability, and signal transduction efficiency.^[^ ^42^ ^]^

For example, a multiplex electrochemical sensor was developed for the detection of six redox‐related biomarkers using electropolymerized MIPs films as selective recognition layers. Each MIPs layer was formed in situ on the electrode surface with the corresponding analyte as the template, enabling highly specific and stable signal generation. The sensor achieved detection limits as low as 20 pmol/L, demonstrated accurate performance in mouse and human blood samples, and successfully distinguished lung cancer patients from healthy individuals based on redox potential (Ehc) analysis. This study highlights the potential of electropolymerization‐based MIPs for high‐throughput, cost‐effective biosensing and liquid biopsy applications in clinical diagnostics.^[^ ^43^ ^]^

**Figure 3:**
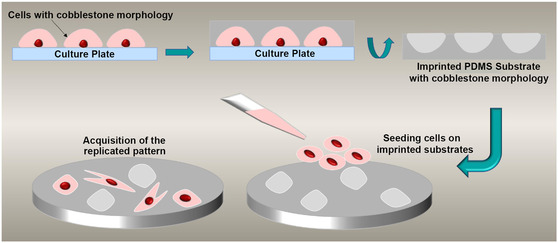
Schematic view of cell imprinting process. Adapted with permission.^[^ ^51^ ^]^

Microcontact stamping, or cell imprinting, is a surface patterning technique that uses entire cells as templates to fabricate substrates containing microscale cavities that mimic the morphology and biochemical surface features of the original cells.^[^ ^44^ ^]^ In a typical process, cells are seeded onto a suitable substrate, like a standard polystyrene dish, and cultured to a confluent state that preserves their native phenotype. These cells are then chemically fixed to stabilize their surface architecture. A premixed solution of a silicone‐based elastomer, such as poly(dimethylsiloxane) (PDMS), is cast over the fixed cell layer to create a negative mold. After curing, the PDMS is peeled off, capturing the microscale and nanoscale features of the cell membrane topography.^[^ ^45^, ^46^ ^]^ This cell‐imprinting strategy enables the fabrication of biomimetic surfaces with cell‐type‐specific recognition, offering valuable applications in cancer diagnostics, stem cell engineering, and tissue regeneration (**Figure** **3**).^[^ ^47^, ^48^, ^49^ ^]^

Material selection plays a crucial role in the success of the imprinting process. Hydrophobic polymers like PDMS are chemically stable at room temperature and possess excellent moldability, optical clarity, and biocompatibility.^[^ ^50^ ^]^ Their ability to physically replicate fine cellular structures down to the nanoscale makes them particularly well‐suited for this technique. In contrast, hydrophilic polymers such as polyacrylamide or methacrylated gelatin (GelMA) can form additional chemical interactions (e.g., hydrogen bonding or ionic interactions) between functional groups on the polymer and those on the cell membrane (e.g., carboxyl and amine groups), potentially enhancing recognition affinity.^[^ ^51^, ^52^ ^]^ However, their susceptibility to swelling or dehydration during processing may compromise structural fidelity. An important consideration is the elastic modulus of the substrate, which influences not only the imprinting resolution but also cellular responses post‐imprinting.^[^ ^51^ ^]^ It is well‐established that cells respond to the stiffness of their extracellular matrix (ECM) through mechanotransduction pathways involving integrins, the actin cytoskeleton, and the linker of nucleoskeleton and cytoskeleton (LINC) complex, ultimately affecting nuclear positioning and gene expression.^[^ ^53^ ^]^ Materials like PDMS are advantageous in this context due to their tunable stiffness, surface modifiability, and gas permeability.^[^ ^54^ ^]^ Despite its advantages, the inherent hydrophobicity of PDMS can lead to poor cell adhesion and limited biological performance on the imprinted surface.^[^ ^55^ ^]^ Moreover, it is difficult to ensure that newly seeded cells will align precisely with the preformed cell‐imprinted cavities. To address these issues, many studies have introduced surface modifications, such as oxygen plasma treatment, chemical grafting, or protein coatings, to enhance hydrophilicity, promote cell attachment, and improve pattern fidelity.^[^ ^56^ ^]^

While with the increasing demand for miniaturized, precise, automated, and high‐throughput systems has driven the development of microfluidic‐assisted MIPs preparation techniques.^[^ ^55^, ^57^, ^58^ ^]^ For example, a novel microfluidic electrochemical chip was developed by integrating MIPs with a two‐electrode configuration and a nanoporous Au‐Ag alloy microwire (NPAMW) modified with electropolymerized MIPs film as the working electrode. This platform achieved ultrasensitive detection of warfarin sodium based on the MIPs “gate effect,” in which the binding of target molecules to the recognition cavities physically blocks the diffusion of electroactive species through the polymer layer, thereby modulating the electrochemical signal, offering a detection limit as low as 8 × 10^−12^ m, well within the clinical requirement range. In addition to warfarin, the same system demonstrated versatility by successfully detecting cyclophosphamide and carbamazepine. When applied to rabbit plasma, the device enabled real‐time therapeutic drug monitoring (TDM) and pharmacokinetic profiling.^[^ ^59^ ^]^

Compared with conventional MIP‐based sensors, the integration of MIPs into microfluidic platforms offers several distinct advantages. First, microfluidics enables precise spatial and temporal control over fluid handling, allowing for highly localized template‐monomer interactions and uniform polymerization conditions, which improve imprinting fidelity and reproducibility.^[^ ^57^ ^]^ Second, the miniaturized architecture of microfluidic chips significantly reduces reagent consumption and analysis time, making the system particularly advantageous for portable, real‐time, and point‐of‐care diagnostics.^[^ ^60^ ^]^ Additionally, continuous flow conditions in microchannels facilitate efficient mass transport, accelerating analyte binding and signal transduction, thereby enhancing sensitivity and response speed.^[^ ^61^ ^]^

Notably, beyond chemical sensing, the synergy between microfluidics and imprinting has also been leveraged in cell‐imprinting applications. In contrast to traditional cell imprinting approaches, where template cells are randomly seeded on substrates, microfluidic systems allow for the precise alignment and positioning of cells during the imprinting process.^[^ ^62^ ^]^ This enhanced control not only improves the uniformity of the imprinted features but also facilitates downstream applications such as spatially guided cell attachment, alignment, and differentiation. For instance, a recent study used a microfluidic chip to create aligned cell‐imprinted PDMS substrates, followed by selective collagen immobilization within the imprinted cavities. This approach significantly improved the trapping efficiency and site‐specific integration of stem cells, ultimately enhancing the differentiation outcomes. Such integration highlights the potential of microfluidic‐assisted cell imprinting for regenerative medicine, where spatial organization and biochemical cues are both critical.^[^ ^62^ ^]^

In addition to innovations in synthetic strategies and device integration, the use of stimuli‐responsive functional monomers has significantly expanded the utility of MIPs in complex biomedical environments.^[^ ^63^ ^]^ These monomers act as both structural and functional components of the polymer network, enabling environmental adaptability tailored to target applications. For example, acrylic acid (AAc), methacrylic acid (MAA), maleic acid (MA), and N,N‐dimethylaminoethyl methacrylate (DMAEMA) are widely used due to their ionizable carboxyl groups, which alter polymer swelling, porosity, or binding affinity under different pH conditions.^[^ ^64^ ^]^ These are particularly useful in tumor‐targeted drug delivery and biosensing under acidic microenvironments. N‐isopropylacrylamide (NIPAm) is the most common thermoresponsive monomer. Poly (N‐isopropylacrylamide) (PNIPAm) exhibits a lower critical solution temperature (LCST) around 32 °C.^[^ ^65^ ^]^ MIPs based on PNIPAm undergo reversible conformational changes near physiological temperature, making them ideal for controlled drug release or reversible capture‐release systems. Azobenzene derivatives undergo reversible cis–trans isomerization under UV or visible light, enabling light‐switchable MIPs systems.^[^ ^66^ ^]^ These are used for optical control over binding site accessibility or photo‐controlled analyte release. These functional monomers enable MIPs to transition from static recognition scaffolds to intelligent, stimulus‐adaptive platforms, capable of responding to the dynamic conditions encountered in physiological settings.

### 2.3 Applications of MIPs

The ability of MIPs to highly selectively adsorb, recognize, and release their target molecules (which can be ions, organic molecules, biological macromolecules, or even whole cells or viruses) gives rise to a wide range of application areas.^[^ ^67^, ^68^ ^]^

**Figure 4:**
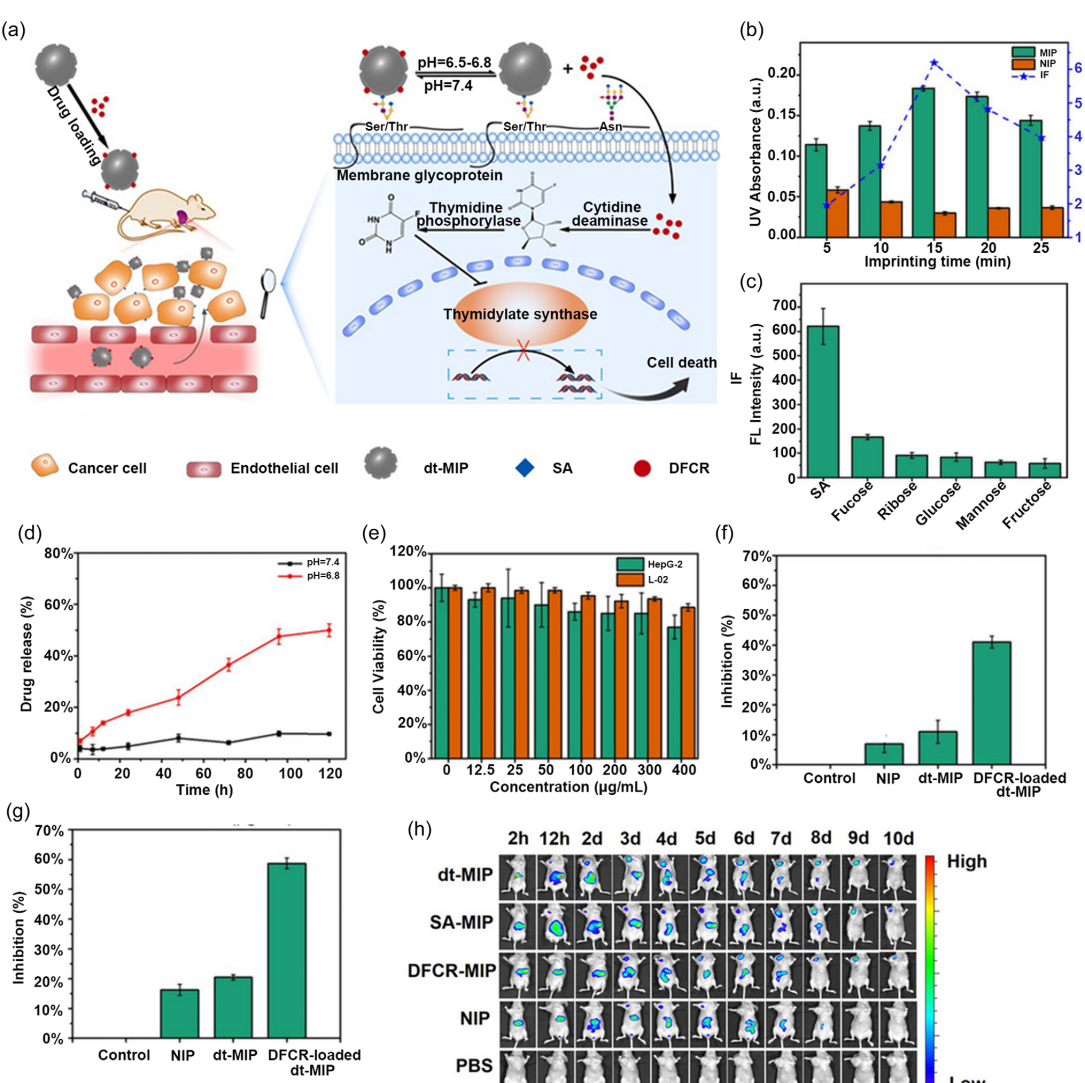
a) Schematic of drug transport and action mechanism in dual‐templated MIP‐based smart targeted delivery system. b) Effect of imprinting time on the binding capability of DFCR‐imprinted and nonimprinted NPs towards DFCR. c) Selectivity of dt‐MIPs prepared with imprinting time of 20 min towards different monosaccharides. d) Release profiles of DFCR in DFCR‐loaded dt‐MIPs at pH 7.4 and pH 6.8. e) Cell viability of liver cancer HepG‐2 cells and normal control L‐02 cells treated with different concentrations of dt‐MIPs. f,g) Inhibition of HepG‐2 cell growth by different materials (200 μg mL^−1^) at 24 h (f) and 48 h (g). h) In vivo fluorescence imaging of HepG‐2 tumor (left upper chest) and liver site (upper abdomen) after intravenous injection of NIR797‐doped dt‐MIPs, SA‐MIPs, DFCR‐MIPs, NIPs and PBS for different times. Adapted with permission.^[^ ^72^ ^]^

MIPs are promising candidates for drug delivery due to their ability to achieve sustained and controlled drug release, making them suitable for potentially treating various medical conditions.^[^ ^69^, ^70^ ^]^ MIP‐based drug delivery systems offer several advantages, including high drug loading capacity (depending on the format) and targeted delivery (**Figure** **4**), which reduce drug dosage and side effects while achieving high drug concentrations in target tissues.^[^ ^71^, ^72^, ^73^ ^]^ Additionally, MIPs can be used to redesign traditional drug formulations, addressing issues such as poor bioavailability and high toxicity of active pharmaceutical ingredients (APIs), thereby improving therapeutic efficacy and expanding their applicability.^[^ ^74^ ^]^

For example, Gu et al. developed a dual‐templated MIPs (dt‐MIPs) system for selective tumor targeting and pH‐responsive prodrug delivery. Using 5′‐deoxy‐5‐fluorocytidine (DFCR) (prodrug) and sialic acid (SA) (tumor marker) as templates, dt‐MIPs nanoparticles were prepared through boronate affinity controllable oriented surface imprinting, a technique that exploits the reversible covalent interaction between boronic acid groups and *cis*‐diol‐containing molecules to achieve highly oriented and selective surface imprinting. The DFCR‐loaded dt‐MIPs accumulated at the tumor site via the enhanced permeability and retention (EPR) effect and further attached to cancer cells via SA‐mediated affinity binding. In the mildly acidic tumor microenvironment (pH 6.5–6.8), the boronate‐DFCR interaction weakens, leading to gradual release of the prodrug from the MIPs matrix. Once internalized by tumor cells, DFCR is enzymatically converted into toxic 5‐fluorouracil (5‐FU) through a two‐step intracellular cascade involving cytidine deaminase and thymidine phosphorylase, thereby inducing cell death via DNA synthesis inhibition. Unlike conventional prodrug strategies that rely on liver‐mediated activation, this MIP‐based delivery system achieves tumor‐specific activation, offering enhanced site selectivity, reduced systemic toxicity, and broader prodrug applicability.^[^ ^72^ ^]^

**Figure 5:**
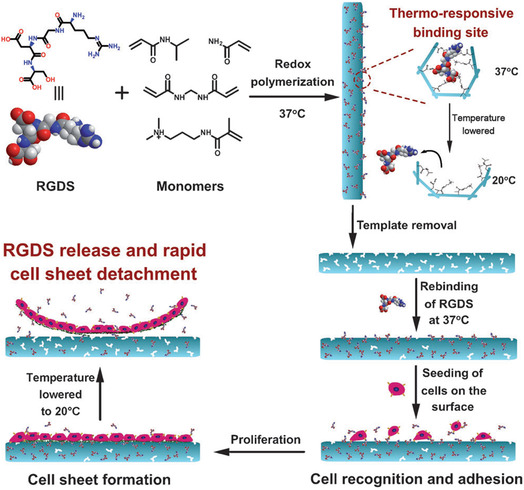
Strategy to introduce the RGDS peptide on a thermo‐responsive cell culture substrate by means of molecular imprinting and the schematic illustration for the cell adhesion and the harvesting of a cell sheet from the system. Adapted with permission.^[^ ^76^ ^]^ Copyright, 2013, WILEY‐VCH Verlag GmbH & Co. KGaA, Weinheim.

The application of MIPs in tissue engineering demonstrates their potential to guide cell behavior by mimicking the ECM or incorporating biochemical signals.^[^ ^13^ ^]^ MIPs can integrate drugs, growth factors, or cell‐adhesion‐promoting peptides into materials using techniques like electrospinning and surface imprinting, enhancing cell proliferation, adhesion, and metabolic activity.^[^ ^75^ ^]^ For example, Pan et al. synthesized a temperature‐responsive hydrogel thin film with specific recognition capabilities for the cell adhesion peptide RGDS using surface imprinting technology. This material enables the selective binding of RGDS to its surface and serves as a substrate for cell sheet culture. The results demonstrated that the material significantly enhances the biocompatibility of the cell culture substrate surface and enables temperature‐controlled adsorption and release of bioactive molecules. This not only effectively promotes cell adhesion and proliferation but also improves the efficiency of cell sheet detachment from the material, addressing long‐standing challenges in cell sheet technology (**Figure** **5**).^[^ ^76^ ^]^

Additionally, MIPs can replicate cell shapes and microenvironmental features through physical cues, such as creating templated surfaces via micro‐contact imprinting, thereby inducing stem cell specific gene expression and morphological changes. These applications highlight MIPs as valuable tools for creating bioactive scaffolds and regulating cellular behavior, with significant potential in tissue engineering.^[^ ^51^ ^]^

**Figure 6:**
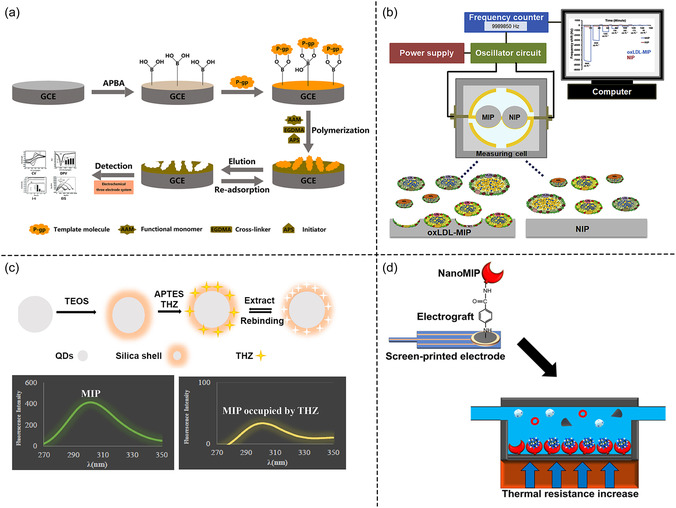
Representative detection mechanisms of MIP‐based biosensors: a) Electrochemical sensor for P‐glycoprotein detection via surface‐imprinted polymerization on a GCE. Adapted with permission.^[^ ^81^ ^]^ Copyright, 2022, Elsevier B.V. All rights reserved. b) QCM sensor for oxLDL based on mass‐sensitive frequency shift induced by MIP‐analyte binding. Adapted with permission.^[^ ^83^ ^]^ Copyright, 2020, Elsevier B.V. All rights reserved. c) Fluorescent optical sensor for thioridazine using quantum dot‐embedded MIPs with fluorescence quenching upon analyte recognition. Adapted with permission.^[^ ^87^ ^]^ Copyright, 2018, Elsevier B.V. All rights reserved. d) Thermal sensor for SARS‐CoV‐2 antigen using NanoMIPs on screen‐printed electrodes with signal readout via heat‐transfer resistance changes. Adapted with permission.^[^ ^89^ ^]^

Lastly, MIPs have become versatile recognition elements in the development of biosensors, enabling highly specific and sensitive detection of a wide range of analytes. These sensors operate through diverse transduction mechanisms, including electrochemical, thermal, mass‐sensitive, and optical modes, each converting the molecular recognition event into a measurable physical signal (**Figure** **6**).^[^ ^77^ ^]^

Among these, electrochemical sensors are the most extensively developed and applied MIP‐based platforms.^[^ ^78^ ^]^ They function by detecting electrical changes induced by the binding of target analytes to the MIPs layer, and can be classified into amperometric, potentiometric, conductometric, and impedimetric types depending on the nature of the measurable output (e.g., current, potential, conductivity, impedance).^[^ ^79^ ^]^ Electrochemical MIPs sensors are especially valued for their low detection limits, rapid response, and adaptability to miniaturization, making them ideal for portable diagnostics.^[^ ^80^ ^]^ For example, a molecularly imprinted electrochemical sensor was developed for the selective detection of P‐glycoprotein (P‐gp) using surface imprinting strategy. The sensor was fabricated on a glassy carbon electrode (GCE) and exhibited outstanding electrochemical performance under physiological conditions. It demonstrated two linear detection ranges: 1.0 × 10^−^^10^ to 1.0 × 10^−^^1^ μg mL and 0.5 to 10 μg mL, with an impressive limit of detection (LOD) of 2.233 × 10^−^^11^ μg mL^−1^. The platform also offered a rapid response time, high selectivity, and good stability, maintaining functionality after 1 week of storage at 4 °C in PBS and allowing up to three reuses with minimal performance loss (RSD <5%). When tested in 1% plasma matrix, the sensor outperformed a conventional ELISA kit in terms of wider detection range, faster assay time, higher accuracy, and lower cost, underscoring its practicality and translational potential for detecting high‐molecular‐weight biomarkers in complex biological samples.^[^ ^81^ ^]^

Mass‐sensitive sensors, such as quartz crystal microbalance (QCM) or surface acoustic wave (SAW) devices, detect changes in mass caused by analyte binding through frequency shifts in an oscillating system.^[^ ^82^ ^]^ These sensors benefit from MIPs’ robust surface binding and selective capture ability, allowing for real‐time, label‐free monitoring of biomolecules with high precision.^[^ ^78^ ^]^ They are particularly effective in affinity assays, molecular interaction studies, and environmental monitoring. For example, a MIPs QCM sensor was developed for the detection of oxidized low‐density lipoprotein (oxLDL) in serum. The MIPs thin film exhibited excellent selectivity, showing minimal cross‐reactivity to similar lipoproteins such as LDL and high‐density lipoprotein (HDL), and negligible response to very‐low‐density lipoprotein (VLDL) and human serum albumin (HSA). The sensor demonstrated a dynamic detection range of 86–5600 μg dL^−1^, fully covering clinically relevant concentrations, with a rapid response time of 10 min, significantly faster than the 210 min required for standard ELISA kits. It also achieved high recovery accuracy (92–107%) and reproducibility (1–8% coefficient of variation). These features underscore the promise of MIP‐based QCM sensors for rapid, label‐free, and clinically applicable detection of sensitive cardiovascular biomarkers.^[^ ^83^ ^]^

Optical sensors leverage changes in light properties, such as absorbance, fluorescence, or refractive index to report molecular recognition.^[^ ^84^ ^]^ In MIP‐based optical sensors, signal transduction can occur through (i) the inherent optical activity of the analyte, (ii) changes in fluorescence or color when chromophores or fluorophores are embedded within the polymer matrix, or (iii) analyte‐triggered catalytic generation of optical signals.^[^ ^78^, ^85^, ^86^ ^]^ Technologies such as UV/Vis spectroscopy, fluorescence, surface plasmon resonance (SPR), and surface‐enhanced Raman scattering (SERS) are frequently used.^[^ ^78^ ^]^ Optical MIP sensors are noted for their noninvasiveness, high sensitivity, and compatibility with real‐time imaging. For example, a fluorescent MIP‐based optical sensor was developed for the selective detection of thioridazine hydrochloride (THZ), using zinc oxide quantum dots (ZnO‐QDs) as the fluorescent core. The MIP shell was synthesized via a microemulsion method, enabling selective recognition of THZ. Upon increasing THZ concentration, fluorescence quenching of the QDs was observed, forming the basis of detection. The sensor achieved a linear dynamic range of 4–120 nmol L^−1^ and a detection limit as low as 0.43 nmol L^−1^, demonstrating excellent sensitivity. Additionally, it showed high selectivity, low relative standard deviations (3.1–4.9%), and successful recovery in serum samples (97–105%), making it a fast, low‐cost, and clinically applicable tool for neuroleptic drug monitoring.^[^ ^87^ ^]^

Thermal sensors detect heat exchange or enthalpic changes associated with molecular binding events. MIPs used in thermal sensors contribute by providing high binding specificity and reproducibility.^[^ ^88^ ^]^ For example, a MIP‐based thermal sensor was developed for the rapid and ultrasensitive detection of SARS‐CoV‐2 antigens. In this system, MIPs were synthesized using a short peptide fragment (10 amino acids) from the SARS‐CoV‐2 spike protein as the template and were subsequently electrografted onto screen‐printed electrodes for integration into a heat transfer method (HTM)‐based assay. The sensor demonstrated exceptional sensitivity, with detection limits as low as 9.9 fg/mL for the alpha variant and 6.1 fg/mL for the delta variant, far surpassing most commercial rapid antigen tests. Notably, the device provided a rapid response time of 15 min and maintained performance under challenging pH (5.5–8.5) and temperature conditions (up to 121 °C).^[^ ^89^ ^]^

## 3 MIPs for Bone Turnover Biomarkers Detection

### 3.1 Metabolism and Biomarkers in Bone Turnover

**Figure 7:**
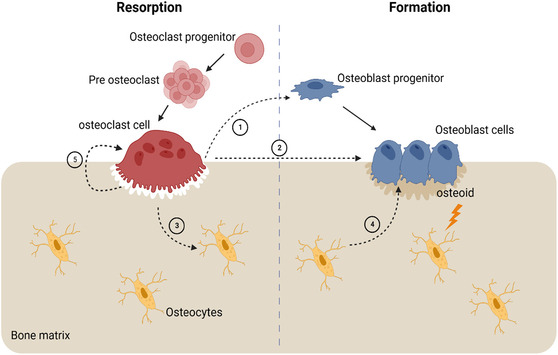
Coupling mechanism of the bone starting with osteoclast cells signaling cells of osteoblast lineage cells in the canopy, osteoblast progenitors, (1) reversal cells in the bone surface, and osteoblast cells (2); it also signals the osteocytes in the bone matrix (3) which signal the osteoblast cells (4); physical changes in the bone are also signaled to the osteoblast cells to secrete and form the appropriate amount of bone matrix. Adapted with permission.^[^ ^182^ ^]^

In the dynamic equilibrium system of bone formation, the basic multicellular unit (BMU, primarily composed of osteoblasts and osteoclasts) plays a pivotal role.^[^ ^90^ ^]^ The continuous self‐renewal process, in which osteoclasts resorb old bone and osteoblasts form new bone, is referred to as bone turnover. Bone turnover is an ongoing process that repairs damage and maintains bone strength (**Figure** **7**).

The BMU sustains the dynamic balance of bone turnover through multiple signaling pathways. Osteoblasts, derived from mesenchymal stem cells, are the main executors of bone formation, promoting mineralization by secreting type I collagen and noncollagenous proteins such as osteocalcin and alkaline phosphatase.^[^ ^91^ ^]^ Their activity is regulated by several key signaling pathways, including: (i) the Wnt/β‐catenin pathway, which activates osteogenic genes like Runx2 and osteocalcin to drive differentiation and bone formation; (ii) the BMP pathway, which regulates osteogenic gene expression via Smads; and (iii) the IGF‐1/PI3K/Akt pathway, which enhances osteoblast proliferation and survival.^[^ ^92^ ^]^

Conversely, osteoclasts, formed through the differentiation and fusion of mononuclear progenitor cells, degrade bone matrix and release stored minerals. This process is controlled by pathways such as (i) the RANK/RANKL/OPG pathway, where RANKL promotes osteoclast differentiation while OPG competitively inhibits it; (ii) the NF‐κB pathway, which activates osteoclast function; and (iii) the macrophage colony‐stimulating factor (M‐CSF) pathway, which supports osteoclast generation and survival.^[^ ^93^ ^]^

Throughout bone turnover, various bone turnover markers (BTMs) are released into the bone microenvironment and accumulate in blood or urine. These markers are classified into bone formation markers [including alkaline phosphatase (ALP), procollagen type 1N‐terminal propeptide (P1NP), procollagen type 1C‐terminal propeptide (P1CP), and osteocalcin (OC)] and bone resorption markers [such as hydroxyproline (HOP), pyridinoline (Pyr), deoxypyridinoline (DPD), L‐Hydroxyproline (L‐HYP), C‐terminal telopeptide of type 1 collagen (CTX‐1), N‐terminal telopeptide of type 1 collagen (NTX‐1), tartrate‐resistant acid phosphatase 5b (TRAP 5b), and interleukin‐6 (IL‐6)].^[^ ^94^ ^]^ These markers provide insights into bone metabolic activity and are valuable for clinical assessments. **Table** **2** details the basic functions of BTMs mentioned above, sources and common detection methods.

**Table 2:** Classification, functions, sources, and detection methods of BTMs.

BTMs	Description	Sources	Detection methods	References
*Bone formation biomarkers*				
ALP	An enzyme secreted by osteoblasts during mineralization, hydrolyzing phosphate esters to promote calcification and reflect overall bone formation activity.	Serum	Enzyme‐linked immunosorbent assay (ELISA), chemiluminescence	[203]
BALP	A specific isoform of ALP found in bone tissue, predominantly in osteoblasts, directly reflecting bone formation activity.	Serum	ELISA, chemiluminescence	[204]
P1NP	A peptide released during type I collagen synthesis, serving as a marker of bone matrix production and dynamic bone turnover rate.	Serum	ELISA, chemiluminescence	[205]
P1CP	A peptide released during type I collagen synthesis, reflecting early stages of bone matrix synthesis and osteoblast activity.	Serum	ELISA	[206]
OC	A small protein secreted by osteoblasts, dependent on vitamin K for calcium binding, involved in regulating calcium and phosphorus in bone tissue.	Serum, Urine	ELISA, radioimmunoassay (RIA)	[207]
*Bone resorption biomarkers*				
HOP	An amino acid metabolite released during type I collagen degradation, serving as a nonspecific marker of bone resorption and also found in soft tissue metabolism.	Urine	HPLC	[208]
Pyr	A crosslinking molecule in collagen fibers that provides mechanical strength, released during bone matrix degradation, and also present in cartilage degradation.	Urine	HPLC, ELISA	[209]
DPD	A specific metabolite of collagen crosslinks derived solely from bone tissue, reflecting bone resorption activity with high specificity.	Urine	HPLC, ELISA	[210]
L‐HYP	A hydroxyproline stereoisomer and collagen metabolite, reflecting collagen degradation and bone resorption.	Serum, Urine	ELISA, HPLC, colorimetric assay	[211]
CTX‐1	A crosslinked carboxy‐terminal fragment released during type I collagen degradation, a highly sensitive marker of bone resorption used in dynamic bone metabolism assessment.	Serum, Urine	ELISA, chemiluminescence	[212]
NTX‐1	A crosslinked amino‐terminal fragment released during collagen degradation, another important marker widely used for assessing bone resorption and monitoring antiresorptive therapy.	Serum, Urine	ELISA, chemiluminescence	[213]
TRAP 5b	A specific isoform of acid phosphatase secreted by osteoclasts, whose activity correlates with osteoclast number and activity, reflecting bone resorption.	Serum	ELISA	[214]
IL‐6	A proinflammatory cytokine associated with bone resorption and osteoclast activation, reflecting inflammation‐induced bone turnover.	Serum	ELISA, chemiluminescence	[215]

Despite the widespread application of the aforementioned methods in clinical detection of bone biomarkers, they present notable limitations. ELISA and chemiluminescence often rely on costly reagents and specialized instruments, restricting their accessibility in resource‐limited settings. Furthermore, these methods demand strict operational conditions and are time‐consuming, making them less efficient for high‐throughput or point‐of‐care testing.^[^ ^95^ ^]^ HPLC, while offering high precision, requires complex sample preparation, extended processing times, and significant operational costs, limiting its practical utility in routine clinical workflows. Additionally, many of these approaches depend on fragile biological components such as antibodies or enzymes, which are prone to degradation under nonoptimal conditions. These shortcomings highlight the urgent need to develop innovative biomarker detection systems that are cost‐effective, robust, and adaptable to diverse clinical environments.^[^ ^96^ ^]^

### 3.2 Advances in MIP‐Based Sensors for BTMs Detection

To address the limitations of current BTMs detection systems, MIPs integrated into various sensor surfaces and signal transducers offer a promising alternative.^[^ ^97^ ^]^ These MIP‐based sensors enable the detection and quantification of biomarkers and biochemical substances in biological fluids such as blood, serum, urine, and saliva. More importantly, MIP‐based sensors provide a cost‐effective solution for monitoring physiological functions. They are easier to operate with minimal training, making them accessible for use in a broader range of settings. Furthermore, unlike biosensors functionalized with antibodies or other conjugates, the incorporation of MIPs significantly reduces production costs. With advancements in MIPs for biosensors, BTMs, such as CTX‐1 and L‐HYP, as well as inflammatory BTM like IL‐6, can be imprinted and utilized for the detection, paving the way for innovative, efficient diagnostic approaches.

Afsarimanesh et al. developed a CTX‐1 sensor by using precipitation polymerization to synthesize the CTX‐1 MIPs. The resulting MIPs particles were immobilized onto the biosensing area of an interdigital sensor using a self‐assembled monolayer (SAM) of acrylic resin, followed by a dip‐coating process to create a uniform MIPs layer.^[^ ^15^ ^]^ The sensor performance was evaluated in real serum samples using a complex nonlinear least squares (CNLS) single‐frequency measurement, demonstrating high precision and achieving detection limits comparable to standard ELISA assays (down to 0.09 ng mL^−1^), but offering a simpler, faster, and more cost‐effective method for CTX‐1 detection, highlighting its potential for practical applications in clinical diagnostics.

**Figure 8:**
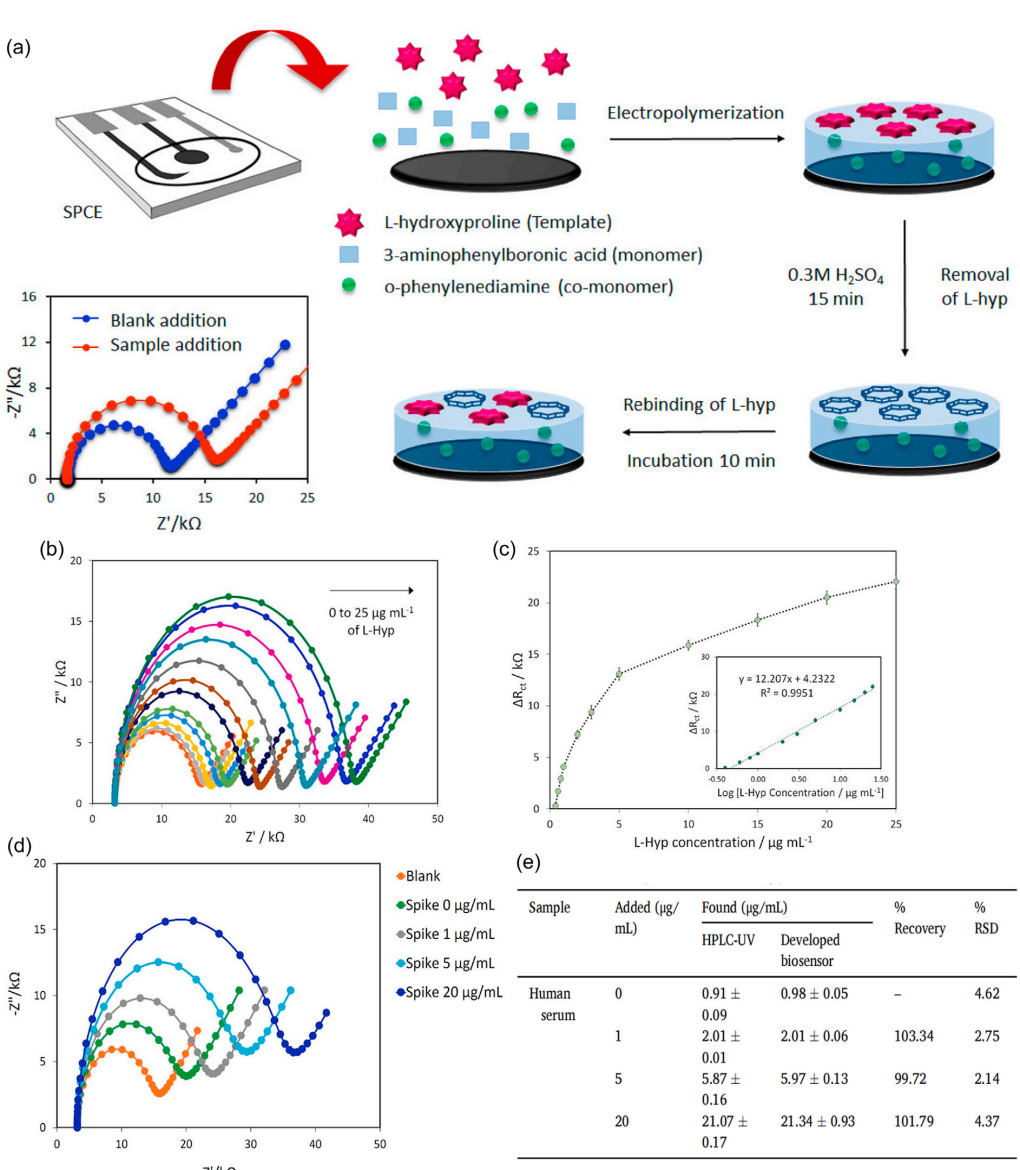
a) Schematic illustration of the fabrication and operation of MIPs/screen‐printed carbon electrode (SPCE) biosensor. b,c) The impedimetric responses of the MIPs/SPCE after incubating with various L‐HYP concentrations of 0.4, 0.6, 0.8, 1, 2, 3, 5, 10, 15, 20, and 25 μg mL^−1^. The plot of charge transfer resistances and concentrations and inset is the plot of ΔRct against logarithmic of L‐HYP concentration. d) Nyquist plots of the MIPs/SPCE biosensor for the analysis of human serum samples with differently spiked L‐HYP concentrations (0, 1, 5, and 20 μg mL^−1^) using the optimal conditions. e) Comparison of the achieved results between the proposed and standard HPLC‐UV method for the quantification of L‐HYP (*n* = 3). Adapted with permission.^[^ ^98^ ^]^ Copyright, 2021, Elsevier B.V. All rights reserved.

Jesadabundit et al. introduced an enzyme‐free electrochemical impedimetric biosensor based on MIPs for the sensitive and selective detection of L‐HYP. The MIPs was co‐electropolymerized using 3‐aminophenylboronic acid (3‐APBA) and o‐phenylenediamine (o‐PD) as monomers in the presence of L‐HYP. Changes in charge transfer resistance (Rct) due to L‐HYP binding were used for detection. The biosensor demonstrated high sensitivity (detection limit: 0.13 μg mL^−1^; quantification limit: 0.42 μg mL^−1^) and selectivity, achieving a dynamic range of 0.4–25 μg mL^−1^. It was successfully applied to human serum samples without pretreatment, providing a simple, portable, and cost‐effective tool for early‐stage bone disease diagnosis (**Figure** **8**).^[^ ^98^ ^]^

Radfar et al. developed a highly sensitive and specific detection platform for IL‐6 based on magnetic MIPs (MMIPs). The study used an epitope peptide of IL‐6 (NH2‐VPPGEDSKDVAA‐COOH) as the template molecule. Magnetic nanoparticles (MNPs) were surface‐modified with the epitope template and APMA, followed by polymerization around the modified MNPs. The template molecule was then removed from the polymer matrix to obtain MMIPs with specific binding. To enable electrochemical detection, the synthesized MMIPs solution was drop‐cast onto the surface of a gold electrode (SPGE).^[^ ^99^ ^]^ Characterization results showed that MMIPs had a total hydrodynamic size of 170 nm with a magnetic core encapsulated by a polymer shell ≈5 nm thick. Electrochemical tests revealed that MMIPs exhibited excellent affinity (dissociation constant KD = 0.25 pM in buffer, 1.6 pM in serum), sensitivity (detection limit of 0.00038 pM), and a broad linear detection range (up to 0.38 pM). Moreover, MMIPs demonstrated superior selectivity compared to existing sensors based on antibodies or aptamers, with an imprinting factor (expressed as ratio between the binding to the MIPs and the binding to the NIP) of >4, highlighting their great potential for IL‐6 detection.

From the above, it transpires that the key advantage of MIPs for detecting BTMs lies in enabling rapid and highly precise analysis, which is crucial for the early diagnosis, real‐time monitoring, and personalized treatment assessment of bone metabolism disorders. To further illustrate the practical benefits of MIP‐based detection, we have summarized representative studies in **Table** **3**, comparing MIP‐based sensors with conventional analytical method, ELISA.

**Table 3:** Comparative performance of traditional and MIP‐based methods for selected BTMs.

BTM	Traditional method	LOD (traditional)	Detection time (traditional)	MIP sensor method	LOD [MIPs]	Detection time [MIPs]	References
CTX‐1	ELISA	0.0503 ng mL^−1^	2–3 h	Electrochemical sensor	0.09 ng mL^−1^	7 min	[15]
L‐HYP	ELISA	0.066 μg mL^−1^	2–3 h	Electrochemical sensor	0.13 μg mL^−1^	15 min	[98]
IL‐6	ELISA	0.28 pg ml^−1^	2–3 h	Electrochemical sensor	9.0 pg mL^−1^	15 min	[99]

As shown in Table 3, MIP‐based sensors typically achieve sensitivities comparable to conventional analytical techniques such as ELISA. However, their most compelling advantages lie in the significantly shortened assay time, which is highly desirable in point‐of‐care and high‐throughput testing scenarios.

ELISA operates by utilizing antibodies immobilized on a solid surface to capture target antigens, followed by signal amplification through enzyme‐linked secondary antibodies.^[^ ^100^ ^]^ While this method offers high specificity, it heavily relies on the structural integrity and biological activity of antibodies, which are sensitive to environmental conditions.^[^ ^101^ ^]^ Compared to biosensors based on biological recognition elements such as antibodies or aptamers, MIPs offer distinct advantages in terms of cost, stability, and robustness.^[^ ^102^ ^]^ Antibodies, while highly specific, are expensive to produce, prone to denaturation, and require strict cold‐chain storage and transport. In contrast, MIPs are synthetic polymers that can be mass‐produced at low cost, are chemically inert, and remain functional under harsh physical and chemical conditions, including extreme pH, temperature, and organic solvents.^[^ ^103^ ^]^

For example, the insulin sensor developed by Ayankojo et al., which used a molecularly imprinted cryogel combined with carboxylated multiwalled carbon nanotubes. The sensor exhibited high selectivity and an ultralow LOD of 33 fM and, notably, retained stable sensing performance after 10 weeks of dry storage at room temperature. In contrast, an ELISA assay based on antibodies would typically lose bioactivity under such storage conditions due to protein denaturation and degradation, requiring refrigeration or freezing for stability.^[^ ^104^ ^]^ This example clearly illustrates how MIPs not only offer high analytical performance but also enable long‐term, low‐maintenance storage, making them particularly attractive for diagnostic applications in decentralized, low‐resource, or mobile testing environments. These cumulative advantages underscore the potential of MIPs as durable, economical, and scalable alternatives to traditional biosensors and strongly support their future application in real‐world clinical diagnostics for BTMs.

### 3.3 Challenges and Clinical Translation in Biomarkers Detection

Despite the significant progress in the development of MIP‐based sensors for BTMs, their clinical translation remains constrained by several critical challenges.

#### 3.3.1 Template Selection and Imprinting Fidelity

A key technical hurdle lies in selecting appropriate template molecules. Since most BTMs are proteins with large molecular weights and conformational complexity, direct imprinting often suffers from low fidelity and accessibility of recognition sites. To address this, researchers have explored the use of peptide epitopes, structural analogs, or dummy templates, which improve polymerization efficiency and binding accessibility.^[^ ^105^, ^106^, ^107^ ^]^ However, these approaches must be carefully optimized to maintain biological relevance and target selectivity.

#### 3.3.2 Nonspecific Adsorption and Matrix Interference

In complex biological fluids such as serum or urine, MIPs face interference from abundant nontarget biomolecules that may nonspecifically adsorb to the sensor surface. This nonspecific adsorption can significantly degrade analytical performance by increasing background noise and reducing specificity.

To overcome this, surface engineering strategies have been introduced. Antifouling strategies involve applying a hydrophilic, inert protective layer on the MIPs surface to repel nonspecific biomolecular adsorption. Common antifouling materials include polyethylene glycol (PEG), zwitterionic polymers such as poly(sulfobetaine methacrylate) (polySBMA) or poly(carboxybetaine methacrylate) (polyCBMA), and hydrophilic polymer brushes.^[^ ^108^, ^109^ ^]^ These coatings form a hydration barrier that sterically and electrostatically resists protein fouling, thereby enhancing signal‐to‐noise ratio and preserving sensor selectivity in protein‐rich samples.^[^ ^110^ ^]^ Another strategy focuses on optimizing the chemical environment of the recognition surface to improve target binding and minimize background signals.^[^ ^111^ ^]^ This involves tuning surface charge, polarity, or introducing specific functional groups that facilitate electrostatic, hydrophobic, or hydrogen‐bonding interactions with the target molecule.

Polydopamine (PDA) has emerged as a unique antifouling component due to its rich catechol and amine functionalities, which form a stable hydration layer through extensive hydrogen bonding.^[^ ^112^ ^]^ This hydration layer effectively resists the nonspecific adsorption of proteins and other macromolecules. Additionally, PDA coatings possess a neutral surface charge and flexible structure, further reducing electrostatic and hydrophobic interactions that typically promote biofouling.^[^ ^113^ ^]^ These characteristics make PDA a valuable material for enhancing the selectivity and biointerface performance of MIP‐based biosensors.

For example, Zhang et al. developed an electrochemical biosensor by using C‐reactive protein (CRP) as the template molecule and integrating conductive graphdiyne (GDY) nanosheets with PEG‐based antifouling layers. Dopamine was used as the functional monomer to form imprinted sites via hydrogen bonding and multipoint electrostatic interactions with CRP. The resulting PDA‐MIPs not only provided high specificity but also contributed to fouling resistance. The additional PEG coating further suppressed nonspecific adsorption from serum proteins. The synergistic effect of PDA, PEG, and GDY enabled a wide linear detection range (10^−^^5^–10^3^ ng mL^−1^), an exceptionally low LOD (0.41 × 10^−^^5^ ng mL^−1^), and high signal stability in complex serum samples.^[^ ^114^ ^]^ This study demonstrates how combining antifouling interfaces with tailored surface chemistry can significantly enhance the performance and reliability of MIP‐based biosensors in clinical applications.

#### 3.3.3 Single‐Analyte Limitation

MIPs are often designed for detecting a single biomarker. However, bone metabolism involves multiple interconnected BTMs, and assessing only one may not yield comprehensive diagnostic value. Future efforts should focus on developing multiplexed platforms, such as MIPs arrays, integrated microfluidic chips, or multichannel electrochemical systems, enabling simultaneous detection of multiple targets for improved clinical insight.

#### 3.3.4 Standardization and Reproducibility

For MIP‐based sensors to be translated into clinical practice, consistent performance across different fabrication batches is essential. Variability in polymer composition, imprinting conditions, or template removal may lead to sensor‐to‐sensor differences. To address this, automated fabrication, microfluidic‐assisted polymerization, and scalable electropolymerization techniques are being explored to improve quality control and manufacturing reproducibility.^[^ ^115^ ^]^

## 4 MIPs for Osteogenic Therapeutic Applications

### 4.1 Current Osteogenic Treatment Methods

Recent advancements have emphasized both pharmacological interventions and biomedical materials to promote osteogenic differentiation.^[^ ^116^ ^]^ **Table** **4** and **5** summarize the most common strategies used to promote osteogenesis, providing brief descriptions of each subclassification's therapeutic mechanism and highlighting the advantages associated with each approach.

**Table 4:** Pharmacological strategies for promoting osteogenesis: mechanisms and advantages.

Classification	Name	Therapeutic mechanism	Advantages	References
Osteogenesis activator	BMPs (BMP‐2, −7, −6, −9)	BMPs bind to type I and type II serine/threonine kinase receptors, activating Smad signaling to stimulate osteoblast differentiation and matrix mineralization.	Highly osteoinductive, stimulate bone formation even in nonbony sites.	[216]
	Small Molecules Enhancing Osteogenic Signaling, e.g., Statins	Statins upregulate BMP‐2 by activating the mevalonate pathway and inhibiting HMG‐CoA reductase in osteoblasts.	Statins can provide dual benefit for cardiovascular and bone health, upregulates local osteogenic signaling.	[118]
Bone resorption inhibitor	BPs	BPs inhibit farnesyl pyrophosphate synthase (FPPS) in the mevalonate pathway, leading to osteoclast apoptosis and reduced bone resorption.	Prevent bone loss, commonly used in osteoporosis management, long‐lasting effect.	[119]
	RANKL inhibitors (denosumab)	RANKL inhibitors binds to RANKL and prevents it from interacting with its receptor (RANK) on osteoclast precursors, thereby inhibiting the formation, function, and survival of osteoclasts.	Potent antiresorptive, reduce fracture risk, effective in high‐turnover bone loss conditions.	[120]
	SERMs	SERMs bind to estrogen receptors (ERα and ERβ) on the osteoclasts, exerting estrogen‐like effects by downregulating osteoclast activity and bone resorption.	Safer alternatives to estrogen replacement therapy for postmenopausal osteoporosis.	[121]
	Calcitonin	Calcitonin binds to calcitonin receptors (CTR) on osteoclasts, activating cAMP signaling, which reduces osteoclast activity and bone resorption.	Calcitonin remains an option for osteoporosis treatment in patients who cannot tolerate BPs or denosumab. Useful for acute pain in vertebral fractures, lowers serum calcium, quick‐acting.	[122]
Dual‐action drugs	PTH and PTH analogs	PTH binds to PTH receptor 1 (PTH1R) on osteoblasts, stimulating cAMP/PKA and Wnt signaling to increase bone formation while transiently increasing osteoclast activity.	Enhances bone density and reduces fracture risk, supports bone repair in osteoporosis.	[123]
	Wnt/β‐catenin pathway modulators	Activate Wnt/β‐catenin signaling, promoting osteoblast differentiation and inhibiting osteoclast activity.	The Wnt/β‐Catenin pathway is a central regulator of osteogenesis and bone homeostasis, providing a broad and sustained impact on bone density, strength, and repair. Dual action of promoting bone formation while minimizing bone resorption.	[124]

**Table 5:** Biomedical materials for osteogenesis: mechanisms and advantages.

Classification	Name	Therapeutic mechanism	Advantages	Limitations	References
Ceramic‐based scaffolds	Hydroxyapatite (HA)	HA scaffolds release calcium (Ca^2+^) and phosphate (PO_4_^3−^) ions, which promote mineralization and support bone matrix formation by mimicking natural bone composition.	Excellent biocompatibility; ability to directly bond with bone tissue; supports long‐term bone regeneration.	Brittle and has low mechanical strength, limiting its use in load‐bearing applications. Requires combination with other materials for better structural performance.	[217]
	Tricalcium phosphate (TCP)	Gradually resorbs in vivo, allowing new bone tissue to replace it over time while releasing calcium and phosphate ions.	Balanced degradation rate and bioactivity, supporting natural bone remodeling.	Faster degradation than HA, which may compromise mechanical stability before complete bone regeneration occurs.	[218]
	Bioactive glass (BG)	Forms a hydroxycarbonate apatite (HCA) layer upon contact with body fluids, facilitating strong chemical bonding with bone. Releases bioactive ions (e.g., Si, Ca, P) to enhance osteogenesis and angiogenesis.	High bioactivity; stimulates osteoblast proliferation and enhances vascularization (angiogenesis).	Brittle and difficult to process into 3D scaffolds. High degradation rate may lead to localized pH changes, affecting cell viability.	[219]
	Calcium sulfate (CaSO_4_)	Provides a temporary scaffold, quickly dissolving to allow space for new bone formation. Acts as a carrier for bone growth factors.	Rapid resorption, particularly useful for dental and orthopedic applications where quick bone formation is needed.	Degrades too quickly to provide long‐term support. Weak mechanical properties; mainly used for small defects or as a bone filler.	[220]
Metal‐based scaffolds	Titanium (Ti)	Forms a strong interface with bone through osseointegration, providing long‐term stability for load‐bearing applications.	High mechanical strength, durability, and corrosion resistance; ideal for orthopedic and dental implants.	Nonbiodegradable, requiring surgical removal in some cases. Stress shielding can occur due to high stiffness, leading to bone resorption.	[221]
	Magnesium (Mg) alloys	Gradually degrades and releases Mg^2+^ ions, which stimulate osteoblast differentiation, collagen synthesis, and growth factor expression. Also promotes angiogenesis and immunomodulation.	Similar elastic modulus to natural bone, reducing stress shielding; supports bone regeneration while degrading.	Rapid degradation can cause hydrogen gas formation, affecting local tissue integration. Requires surface modification to control degradation rate.	[222]
Polymer‐based scaffolds	Collagen‐based scaffolds	Provides a natural ECM‐like structure, facilitating cell adhesion, proliferation, and differentiation.	Excellent biocompatibility, minimizing immune responses and supporting osteogenic differentiation.	Weak mechanical properties, making them unsuitable for load‐bearing applications. Often requires reinforcement with ceramics or synthetic polymers.	[223]
	Poly(lactic‐co‐glycolic acid) (PLGA)‐based scaffolds	PLGA can be engineered into porous structures, fibers, or injectable matrices, offering tunable degradation rates to match bone healing.	Highly versatile, allowing for controlled degradation and mechanical tuning for different applications.	Acidic degradation byproducts can cause localized inflammation. Mechanical properties degrade over time, affecting long‐term structural support.	[224]
Composite scaffolds/others Hybrid scaffolds (e.g., HA/collagen, HA/PLGA, HA/metal chitosan‐alginate, etc.)		Combines materials to merge advantages and mitigate weaknesses, improving mechanical strength, degradation control, and osteogenic potential.	Superior properties compared to single‐material scaffolds; tailored mechanical, biological, and degradation characteristics for specific applications.	More complex fabrication processes; material interactions can lead to unpredictable degradation rates or loss of bioactivity.	[225, 226, 227, 228, 229, 230, 231]

#### 4.1.1 Pharmacological Interventions

Pharmacological interventions play a crucial role in osteogenesis by either stimulating bone formation or inhibiting bone resorption Table 4. Osteogenesis enhancers, such as bone morphogenetic proteins (BMPs), promote osteoblast differentiation and matrix mineralization, significantly enhancing bone density.^[^ ^117^ ^]^ Additionally, statins, traditionally used for cholesterol management, have been found to enhance osteoblast activity and promote bone formation in animal models.^[^ ^118^ ^]^

On the other hand, antiresorptive agents such as bisphosphonates (BPs) and RANKL inhibitors (denosumab) reduce osteoclast activity, thereby lowering bone turnover and preserving bone mass.^[^ ^119^, ^120^ ^]^ Selective estrogen receptor modulators (SERMs), such as raloxifene, mimic estrogen's protective effects on bone by reducing bone resorption while maintaining bone density, making them particularly beneficial for postmenopausal osteoporosis.^[^ ^121^ ^]^ For those patients who cannot tolerate BPs or denosumab in the osteoporosis, calcitonin remains to be another good option for similar therapeutic effect, which acts more quickly.^[^ ^122^ ^]^

Some pharmacological agents exert a dual effect by both promoting osteogenesis and inhibiting osteoclast activity, ensuring a balanced bone remodeling process. Parathyroid hormone (PTH) analogs, such as teriparatide, stimulate osteoblast activity while also reducing osteoclast‐mediated bone resorption, leading to net bone gain.^[^ ^123^ ^]^ Similarly, Wnt/β‐Catenin pathway modulators regulate osteoblast differentiation and suppress osteoclastogenesis, enhancing both bone mass and structural integrity.^[^ ^124^ ^]^ These modulators include activators such as antisclerostin antibodies (which block sclerostin to enhance Wnt signaling and increase osteoblast activity) and glycogen synthase kinase‐3β inhibitors (which prevent β‐Catenin degradation to further stimulate osteoblast differentiation). These modulators can also exhibit an inhibiting action such as dickkopf‐related protein 1 inhibitors and secreted frizzled‐related protein inhibitors (which work by blocking Wnt antagonists, thereby enhancing bone formation and reducing osteoporosis risk).^[^ ^125^, ^126^ ^]^ Since the Wnt/β‐Catenin pathway is highly conserved in osteoblast lineage cells, its modulation offers a targeted approach with fewer off‐target effects.^[^ ^127^ ^]^ These dual‐action therapeutic effects could help to achieve a more balanced bone remodeling, which is essential for sustained bone health.

#### 4.1.2 Bioactive Scaffolds

Bioactive scaffolds are essential to bone tissue engineering, providing mechanical support and biochemical signals to facilitate bone regeneration.^[^ ^128^, ^129^ ^]^ These scaffolds mimic the ECMs, guiding cellular behavior and promoting osteogenesis.^[^ ^130^ ^]^ They can be broadly classified into ceramic‐based, metal‐based, polymer‐based, and composite scaffolds, each with distinct properties that influence their clinical applications Table 5 to clearly indicate the corresponding content.

Ceramic‐based scaffolds are widely used due to their biocompatibility and osteoconductivity, making them ideal for bone integration.^[^ ^131^, ^132^ ^]^ However, their inherent brittleness and limited mechanical strength often necessitate reinforcement with other materials.^[^ ^133^ ^]^ Metal‐based scaffolds, particularly titanium and magnesium, are favored in load‐bearing applications due to their high strength, though issues like stress shielding (Ti) and rapid degradation (Mg) need to be addressed.^[^ ^134^ ^]^

Polymer‐based scaffolds offer flexibility and tunable degradation rates, allowing for controlled bioactivity.^[^ ^135^ ^]^ However, their mechanical weakness limits their use in structurally demanding environments. To overcome these limitations, composite scaffolds integrate multiple materials to achieve a balance between mechanical strength, bioactivity, and degradation control.^[^ ^133^, ^136^, ^137^, ^138^, ^139^ ^]^

With the continuous advancements in bone tissue engineering, the limitations of single‐material scaffolds have driven researchers to explore multifunctional bioactive scaffolds. Current research not only focuses on the mechanical properties, degradation behavior, and bioactivity of scaffolds but also aims to optimize their interactions with cells and biomolecules to enhance clinical outcomes and long‐term applicability. To this end, the next generation of bioactive scaffolds is evolving toward greater functionality.

In this context, the introduction of polymer technology has provided bone repair scaffolds with enhanced controllability. Among these, MIPs exhibit great potential due to their ability to form highly specific molecular recognition sites within scaffolds, facilitating controlled drug release, cell behavior regulation, and bone regeneration. The following sections will explore the applications of MIPs in bone tissue engineering, with a particular focus on their role in guiding stem cell differentiation and controlled drug delivery systems.

### 4.2 Advances in MIP‐Based Osteogenic Treatment

In recent years, the integration of advanced polymer technologies into osteogenic scaffolds has opened new possibilities for precision and efficacy in bone regeneration. Among these, MIPs stand out for their ability to create highly specific binding sites within polymer scaffolds. MIPs designed with drugs or bioactive factors as templates enable stimuli‐responsive drug release and significantly enhance osteogenesis, while MIPs constructed using mature osteoblasts as templates can induce the directional differentiation of stem cells. By leveraging these unique properties, MIPs enhance scaffold performance through controlled drug delivery and precise modulation of cellular behavior, supporting bone formation. The following sections will explore two key applications of MIPs in bone tissue engineering: their potential in cell imprinting technology to guide stem cell differentiation toward osteogenesis and their role in scaffold‐embedded drug delivery systems.

#### 4.2.1 Cell Imprinting Technology: Guiding Stem Cell Differentiation Toward Osteogenesis

ECM is a complex network structure composed of hydrated macromolecular proteins and polysaccharides. It forms the acellular microenvironment of tissues and is considered an ideal source of carrier scaffolds as a natural biomaterial in tissue engineering.^[^ ^140^ ^]^ Its unique chemical, mechanical, and physical properties provide critical support for cell adhesion, migration, proliferation, and differentiation. In fact, various environmental variables within the ECM—physical, chemical, and mechanical—can promote stem cell proliferation and differentiation into specific cell lineages.^[^ ^141^ ^]^

By mimicking the physicochemical conditions of natural ECM, including its proteins, surface morphology, and architecture, culture substrates can stimulate stem cell development, offering an environment conducive to adhesion, growth, migration, and differentiation.^[^ ^142^ ^]^ Technologies based on molecular imprinting, such as cell imprinting and topographic engineering, have further advanced this approach. By replicating the composition, mechanical properties, morphology, and 3D geometry of the ECM, these technologies enable precise regulation of cell behavior, thereby enhancing the potential for tissue and organ regeneration. In particular, these biomimetic scaffold designs demonstrate significant advantages in inducing stem cell differentiation.^[^ ^51^, ^143^ ^]^

Polydimethylsiloxane (PDMS) is one of the primary polymers used in cell imprinting studies, known for its ability to simulate physiological niches, low production costs, and excellent biocompatibility.^[^ ^62^ ^]^ Research has shown that cell‐imprinted PDMS substrates can effectively guide stem cells to differentiate into specific mature cell lineages.^[^ ^144^ ^]^ However, the hydrophobic nature of PDMS results in weak cell adhesion, and the coexistence of imprinted and nonimprinted cavities can lead to uneven cell spreading. These limitations have posed challenges in earlier studies of cell imprinting technology.^[^ ^145^ ^]^ Consequently, strategies such as plasma treatment, chemical modification, and coating techniques have been developed to improve the efficiency of cell imprinting methods.

**Figure 9:**
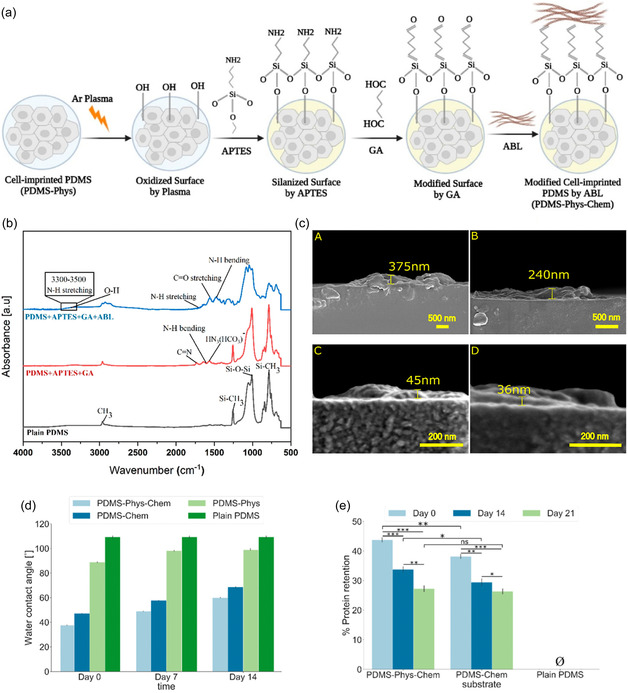
a) Schematic presentation of PDMS surface modification by APTES, GA, and ABL. b) FTIR‐ATR spectra of plain PDMS, plasma‐treated PDMS + APTES + GA, and plasma‐treated PDMS + APTES + GA + ABL at the wavenumber between 500 and 4500 cm^−1^. c) Cross‐sectional FESEM micrographs of (A) 100%, (B) 70%, (C) 30%, and (D) 10% of initial concentration of ABL. d) Surface hydrophilicity analysis of PDMS surfaces as a function of time. Plain PDMS compared with treated groups; PDMS‐Phys (cell‐imprinted PDMS), PDMS‐Chem (flat PDMS with APTES + GA + ABL), and PDMS‐Phys‐Chem (cell‐imprinted PDMS with APTES + GA + ABL) at days 0, 7, and 14 after surface modification. Error bars indicate the standard deviation of the means *n* = 3 samples. e) Micro‐BCA assay for calculation of the amount of attached and retained proteins on the plain PDMS, PDMS‐Chem, and PDMS‐Phys‐Chem substrates on 0, 14, and 21 days. The graph is represented as the mean ± SD. *p‐value of <0.05, **p‐value of <0.01, and ***p value of <0.001 between two groups. *p* value of ≥0.05, Ø represents zero measurement on the plain PDMS. Adapted with permission.^[^ ^146^ ^]^

Babaei et al. developed a method to immobilize the bioactive acidic bone lysate (ABL) on the surface of PDMS substrates after osteogenic cell imprinting. ABL is a biomaterial extracted from bone tissue, typically prepared under acidic conditions, such as using HCl solution. ABL contains various bone‐derived proteins, such as, TGF‐β1 and BMPs. Babaei et al. treated PDMS substrates, with or without osteogenic cell‐imprinted cavities, with an argon plasma, followed by immersion in 2% 3‐aminopropyl triethoxysilane (APTES) solution followed by exposure to glutaraldehyde (GA), which acted as a crosslinking agent to stabilize the chemically modified surface structure. Finally, the substrates were incubated overnight with ABL solution to further modify the surface with its abundant bioactive factors (**Figure** **9**).^[^ ^146^ ^]^ This methodology allowed to successfully immobilize active biomolecules on the PDMS surface. The immobilized proteins demonstrated strong stability, with the possibility of tuning the layer thickness. This latter was optimized to 50 nm, allowing to effectively bind ABL proteins to the substrate while preserving the cell‐imprinted morphology's ability to influence cell fate.

**Figure 10:**
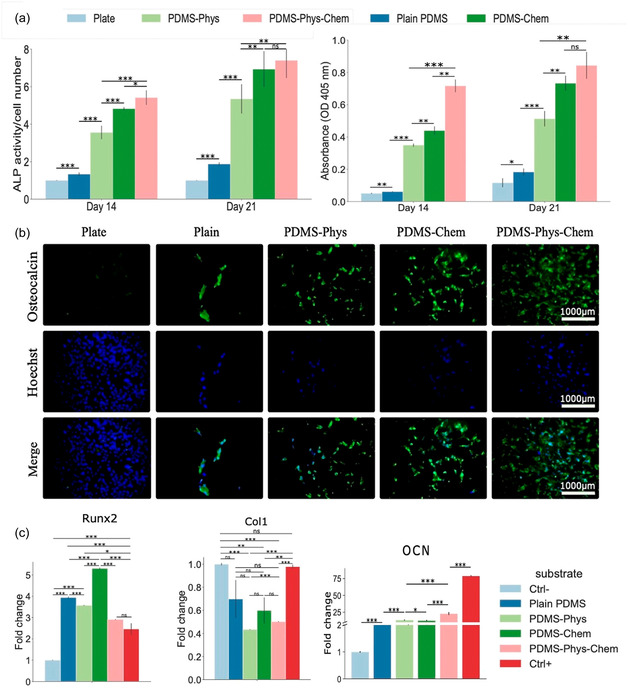
a) ALP activity of ADSCs cultured on the plate, plain PDMS, PDMS‐Phys, PDMS‐Chem, and PDMS‐Phys‐Chem after 14 and 21 days (left). Quantitative analysis of alizarin red S staining of ADSCs grown on different PDMS substrates compared to the plate group after 14 and 21 days (right). b) IF staining of ADSCs cultured on plate, plain PDMS, PDMS‐Phys, PDMS‐Chem, and PDMS‐Phys‐Chem substrates after 21 days. Hoechst staining for the cell nucleus and the FITC‐conjugated antibody for OCN labeling was applied. c) Gene expression of RUNX2, Col1a1, and OCN in ADSCs cultured on the plate (as a negative control), plain, PDMS‐Phys, PDMS‐Chem, and PDMS‐Phys‐Chem substrates after 21 days. (*P < 0.05, **P < 0.01, ***P < 0.001, *p* value ≥0.05, and (ns) indicates a statistically nonsignificant result, *n* = 3). Adapted with permission.^[^ ^146^ ^]^

Adipose‐derived stem cells (ADSCs) were used to evaluate the ability of the combined chemical functionalization and nano‐3D cell imprinting on the PDMS surface to induce stem cell differentiation. ALP activity, calcium release, OCN protein levels, and bone‐specific gene expression were analyzed to determine the direction of stem cell differentiation. The results demonstrated that physical and chemical surface modifications significantly promoted osteogenic differentiation of ADSCs synergistically (**Figure** **10**).

Kamguyan et al. focused their study on the mechanical properties of PDMS substrates by preparing polydimethylsiloxane/hydroxyapatite (PDMS/HA) nanocomposites to investigate how substrate mechanical properties, particularly viscoelastic behavior, influence the differentiation of ADSCs into osteogenic cells. HA powder was mechanically mixed with PDMS to achieve uniform dispersion, and osteogenic cell topographical features were successfully imprinted onto the substrate surface using a cell imprinting method. The study revealed that as the HA content increased (1, 2, and 4 wt%), the mechanical properties of the substrate changed significantly, including variations in surface elastic modulus and viscoelastic behavior.^[^ ^147^ ^]^ Furthermore, the increased HA content also enhanced the hydrophilicity of the PDMS/HA nanocomposites, which further improved cell adhesion and spreading. Through various characterizations (ALP activity, osteocalcin levels, gene expression analysis, etc.), the authors found that the 2 wt% and 4 wt% HA nanocomposite substrates significantly enhanced the osteogenic differentiation of stem cells. This effect was attributed to the physical guidance provided by the imprinted topography, changes in substrate mechanical properties induced by HA, and the osteoinductive signals released by HA nanoparticles (elevating calcium ion and phosphate ion levels), which collectively promoted stem cell differentiation. These findings demonstrate that by tuning the morphology, mechanical properties, and chemical characteristics of PDMS/HA nanocomposites, effective regulation of stem cell differentiation can be achieved, offering new design insights for tissue engineering and regenerative medicine.

Izadi et al. focused their study on uncovering how the physical structure of cell‐imprinted substrates (e.g., topographical features) and the chemical environment (e.g., the addition of β‐carotene, βC) work synergistically to induce the osteogenic differentiation of ADSCs. In this research, ADSCs were seeded onto MG63 osteoblast cell‐imprinted PDMS substrates and cultured in the presence of βC for up to two weeks. The results demonstrated that ADSCs exhibited excellent adhesion and proliferation on the imprinted substrates and successfully differentiated into osteoblast cells, which have potential applications in bone tissue engineering and regenerative medicine.^[^ ^148^ ^]^

Pazooki et al. investigated the mechanism by which cell imprinting and collagen molecule imprinting act as physical stimuli to synergistically promote the differentiation of ADSCs into osteoblasts. Using fixed MG63 osteoblast cells and collagen type I coatings as templates, they successfully achieved dual imprinting of cell shapes and collagen molecules on PDMS substrates. The study found that ADSCs cultured on substrates with both cell and collagen imprints gradually changed their morphology from spindle‐shaped to triangular, indicating a significant cellular response to physical stimulation. Alizarin red staining and calcium assays showed a marked increase in ECM calcium deposition during the 7‐ and 14‐day culture periods. Furthermore, high expression levels of osteogenesis‐related genes, such as osteocalcin, collagen I, and ALP, as well as immunocytochemical detection of bone marker proteins, confirmed the superior efficacy of these substrates in promoting osteogenic differentiation.^[^ ^149^ ^]^ The findings demonstrate that the synergistic effects of cell shape and collagen imprinting on the substrate effectively upregulate osteogenic genes and proteins through physical stimulation, providing a novel strategy for bone tissue engineering.

To further enhance the precision and reproducibility of cell‐imprinted substrates, recent studies have integrated microfluidic technologies into imprinting systems. Microfluidic systems offer several advantages, including precise control over fluid flow, spatial confinement of cells, and continuous nutrient exchange, thus providing a more physiologically relevant and reproducible culture environment.

**Figure 11:**
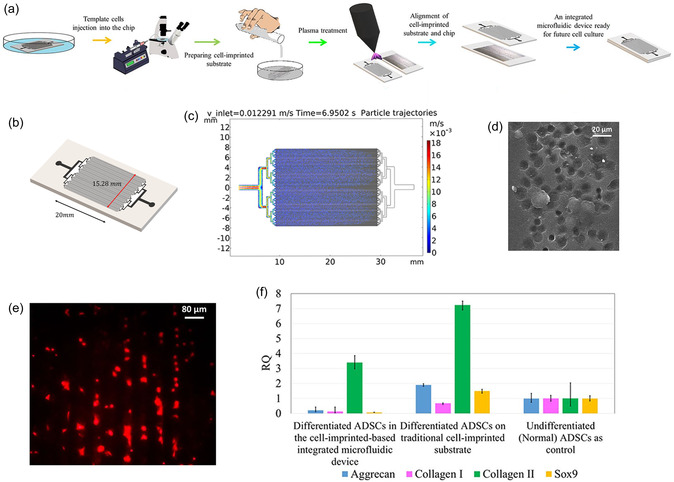
a) Schematic design of the microfluidic chip featuring 128 parallel 40 μm‐wide microchannels, enabling precise flow and cell positioning. b) Fabrication process of the integrated device, including chondrocyte patterning, replica molding, and microchannel alignment. c) Simulated cell trajectories inside the microfluidic chip, highlighting controlled cell positioning and dynamic flow behavior during injection. d) Scanning electron microscopy (SEM) image of the chondrocyte‐imprinted substrate, showing faithful replication of cell morphology. e) Immunofluorescent staining of ADSCs cultured on the imprinted substrate within the microfluidic chip using AlexaFluor488‐conjugated wheat germ agglutinin (WGA), illustrating membrane organization and cell‐surface interactions. f) Quantitative real‐time PCR results showing relative expression of chondrogenic markers (aggrecan, collagen I, collagen II, and Sox9) in ADSCs cultured in the integrated device, compared to traditional imprinting and control groups, confirming enhanced differentiation potential without chemical induction. Adapted with permission.^[^ ^150^ ^]^

A representative example is a recent study in which a chondrocyte‐imprinted microfluidic platform was constructed to enhance the efficiency of ADSC differentiation. In this approach, chondrocytes were first cultured in a microfluidic chip with regularly patterned channels, and their topography was transferred to a silicone substrate via cell imprinting. A second microfluidic chip with matching geometry was then aligned to the imprinted substrate to create an integrated system for controlled cell culture. By aligning the seeded ADSCs precisely with the chondrocyte‐shaped imprints, this method significantly improved the uniformity and efficiency of cell–topography interactions compared to conventional random imprinting (**Figure** **11**).^[^ ^150^ ^]^

To further optimize the dynamic environment, computational fluid dynamics (CFD) simulations were performed to evaluate shear stress distribution and cell trajectory behavior under different flow conditions, enabling accurate prediction of experimental parameters. After 14 days of culture in the device, ADSCs exhibited morphological changes from a fibroblast‐like to a spherical chondrocyte‐like phenotype, even without the addition of biochemical inducers. Immunostaining and gene expression analyses confirmed enhanced chondrogenic differentiation, validating the effectiveness of this combined physical–topographical approach.

Moreover, the dynamic culture environment created by the microfluidic system mimics in vivo fluidic flow, enhancing cellular responses to mechanical cues. The system also requires fewer cells per experiment, making it a cost‐effective and scalable alternative to traditional culture methods.

#### 4.2.2 Scaffold‐Embedded Drug Delivery Systems: Enhancing Osteogenic Efficiency

Scaffold‐embedded drug delivery systems represent a critical innovation in bone tissue engineering, offering precise control over the release of therapeutic agents to promote bone regeneration. MIPs play a pivotal role in this advancement by providing highly selective and stimuli‐responsive drug release capabilities. Through their unique design, MIP‐based scaffolds not only ensure the targeted and controlled delivery of osteogenic drugs but also enhance antibacterial activity and support bone regeneration processes. The following discussion highlights key studies and methodologies, demonstrating the versatility and effectiveness of MIP‐integrated scaffolds in orthopedic applications.

**Figure 12:**
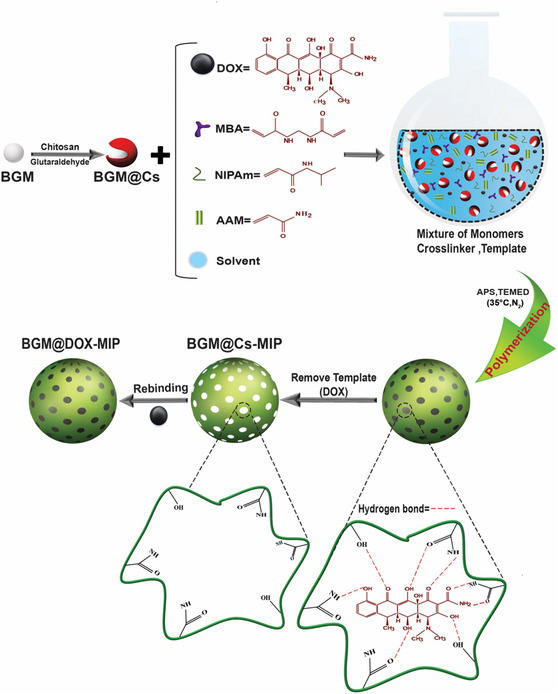
Schematic illustration of the DOX‐imprinted BGM@Cs (BGMs@Cs‐MIPs). Adapted with permission.^[^ ^151^ ^]^ Copyright, 2024, American Chemical Society.

**Figure 13:**
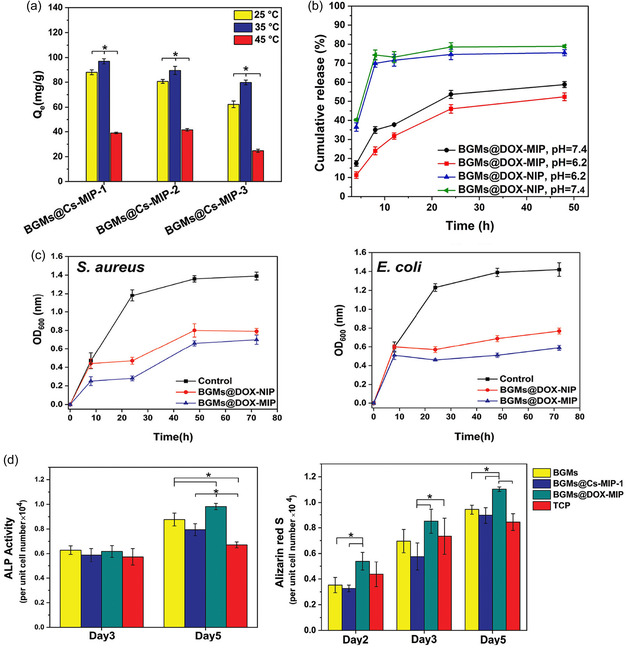
a) Equilibrium adsorption capacity of BGMs@Cs‐MIPs at 25, 35, and 45 °C (pH =7). b) Cumulative release of DOX from BGMs@DOX‐MIPs‐1 and BGMs@Cs‐NIP‐1 at different pH and 37 °C. c) Growth curves of *S. aureus* and *E. coli* incubated with BGMs@DOX‐MIPs and BGMs@DOX‐NIP. All values are reported as means ± SD (*n* = 3). * illustrates a statistically significant difference (*P* < 0.05). d) ALP activity of MG‐63 cells in contact with samples after 3 and 5 days of culture and quantitative results of alizarin red staining after incubation of cells with various samples and control (tissue culture plate, TCP). Adapted with permission.^[^ ^151^ ^]^ Copyright, 2024, American Chemical Society.

Khademi et al. designed and synthesized doxycycline (DOX) molecularly imprinted bioglass microspheres (BGMs) with temperature‐responsive adsorption and controlled release properties for the treatment of postoperative bacterial infections in orthopedics while promoting osteogenesis. BGMs were prepared via a sol‐gel method and then surface‐modified with chitosan (Cs) to generate free amino and hydroxyl groups, thereby reducing surface charge (forming BGMs@Cs). Subsequently, imprinted polymer coatings were synthesized on BGMs@Cs using DOX as the template molecule, resulting in BGMs@Cs‐MIPs. The inclusion of N‐isopropylacrylamide (NIPAm) in the monomer mixture ensure the introduction of temperature‐responsive properties into the final product, but the imprinting process needed to be optimized to be carried out in the same range of body temperature (35 °C) to optimize reaction kinetics and prevent volumetric phase‐transition losses due to temperature fluctuations in vivo (**Figure** **12**).^[^ ^151^ ^]^ Studies revealed significant differences in the equilibrium adsorption capacity (*Q*_e_) of BGMs@Cs‐MIPs under various temperature and crosslinking amounts [indicated by the concentration of methylene‐bisacrylamide (MBA)] (**Figure** **13**a), suggesting its potential for hydrophobicity‐induced controlled drug release in response to temperature increases caused by infections. Further characterization demonstrated that BGMs@DOX‐MIPs exhibited excellent controlled release and cumulative release properties, remarkable antibacterial activity against *Staphylococcus aureus*, and significantly increased ALP activity and calcium mineralization, showcasing its promising application in infection treatment and bone regeneration (Figure 13b–d).

**Figure 14:**
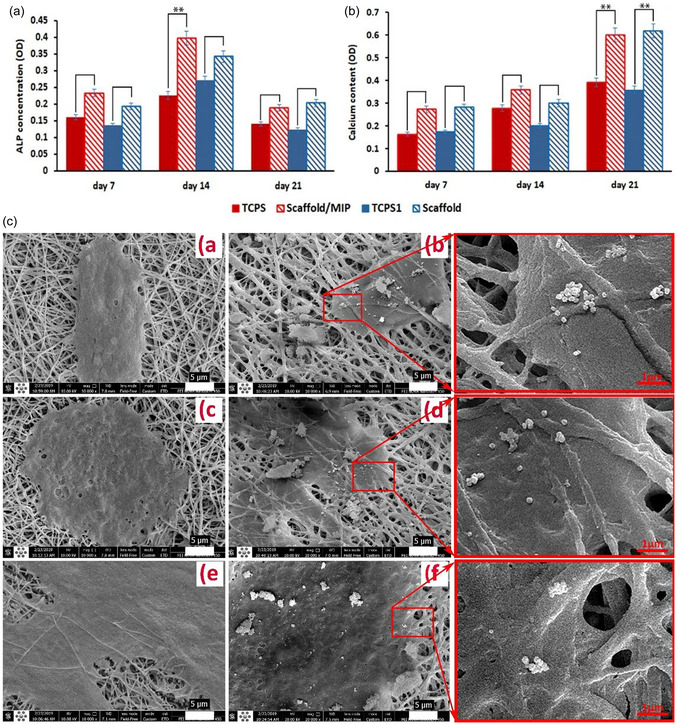
The osteogenic mineralization analysis of two group samples is shown. a) ALP analysis of hMSCs. b) The measured optical density levels of calcium minerals deposited by hMSCs due to osteogenic induction (Dexa‐loaded MIPs NPs besides the PLDLLA/PAAc‐10 nanofibrous scaffold denoted by Scaffold/MIPs, Dexa directly used besides the nanofibrous sample denoted by Scaffold, the cell culture environment containing Dexa‐loaded MIPs NPs denoted by TCPS, and the cell culture medium consisting of Dexa directly without MIPs denoted by TCPS1). c) FE‐SEM micrograph images of Scaffold (a,c,e) and Scaffold/MIPs (b,d,f) after day 7 (a,b), day 14 (c,d), and day 21 (e,f) of cell seeding. Adapted with permission.^[^ ^152^ ^]^ Copyright, 2020, Elsevier B.V. All rights reserved.

Bohlouli et al. synthesized dexamethasone (Dexa)‐loaded MIPs NPs via emulsion polymerization, resulting in MIPs NPs with a high drug loading capacity (*Q* = 57% w/w), which were subsequently integrated in an electrospun nanofibrous composite scaffold composed of poly(l‐lactide‐co‐d,l‐lactide) and poly(acrylic acid) (PLDLLA/PAAc). PLDLLA is widely used in tissue engineering due to its excellent biocompatibility; its poor hydrophilicity and slow degradation rate, however, limit its application in scaffold materials. Bohlouli et al. optimized key parameters in the electrospinning process and successfully achieved small nanofiber diameters, with enhanced hydrophilicity, increased porosity, and fast degradation rates, significantly improving the potential of this material for tissue engineering applications.^[^ ^152^ ^]^ The hydrophilic nature of the PLDLLA/PAAc‐10 scaffold supports greater cell adhesion and subsequent cell proliferation, making this material highly advantageous for cell culture and tissue repair. When coupled with the Dexa‐loaded MIPs NPs into a composite (denoted as Scaffold/MIPs), the scaffold not only enabled controlled drug release but also demonstrated excellent biocompatibility and potential osteoinductive activity, therefore offering broad clinical application potential for bone tissue repair (**Figure** **14**).

Unlike the aforementioned research approach, which utilizes molecular imprinting technology to create cavities or physical topologies with specific binding capabilities for template molecules to achieve molecular detection, drug delivery, or stem cell differentiation induction, Chen et al.'s study combines molecular imprinting technology with sacrificial template methods to focus on controlling the internal pore structure of hydrogels and the chemical modification of the inner pore surfaces with BPs.

BPs are widely used to inhibit bone resorption. When coupled with polymers, BPs not only retain their inhibitory effects on bone resorption but also exhibit specific biological effects, significantly promoting osteogenic differentiation of bone marrow stromal cells (BMSCs) while suppressing the differentiation of macrophages into osteoclasts. However, reported BP‐based scaffolds often rely on dynamic chemical bonds or unstable molecular aggregates, making it challenging to maintain a high‐density functional molecular microenvironment.

**Figure 15:**
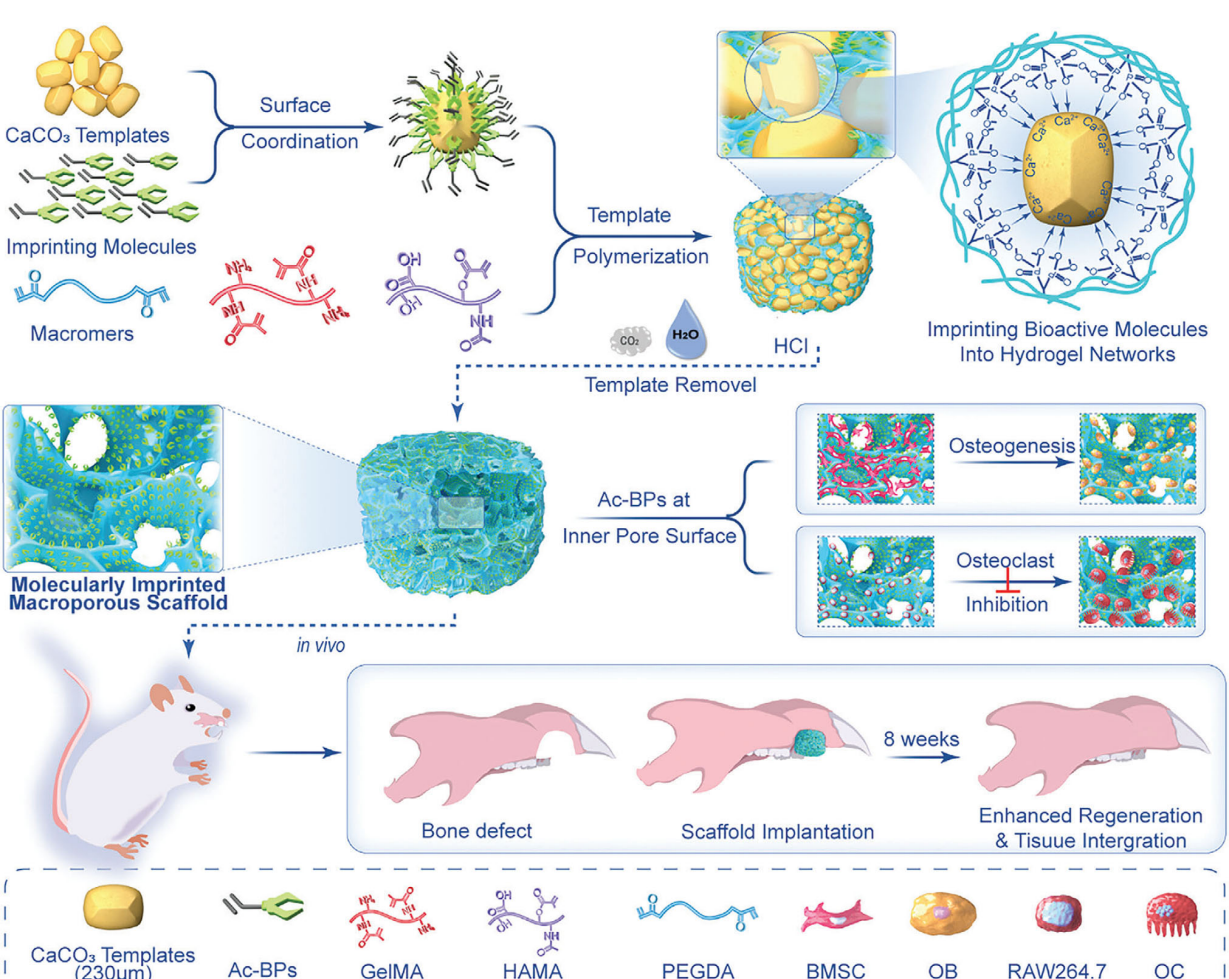
Schematic illustration of GHP‐int‐BP macroporous hydrogel fabrication with inner pore surface modification through the sacrificial template assisted molecular imprinting method. Adapted with permission.^[^ ^153^ ^]^ Copyright, 2024, Wiley‐VCH GmbH.

Chen et al. used eggshell‐derived CaCO_3_ particles as sacrificial templates, anchoring functional acrylated BPs (Ac‐BPs) to the template surface through chelation. These functionalized templates were then crosslinked with a matrix composed of methacrylated derivatives of gelatin (GelMA) and hyaluronic acid (HAMA) and PEG diacrylate (PEGDA). Finally, the CaCO_3_ particles were removed by acid treatment to form hydrogels with interconnected macroporous structures while preserving the chemical modification of functional molecules on the pore walls (GHP‐int‐BP) (**Figure** **15**).^[^ ^153^ ^]^

**Figure 16:**
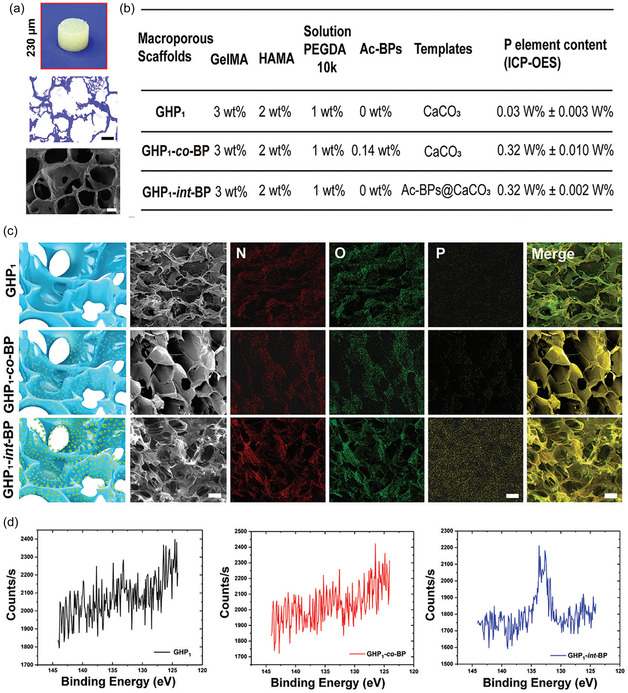
a) Photographs, optical microscope images of crystal violet staining (scale bars: 100 μm) and the internal scanning electron microscope (SEM) observations (scale bars: 50 μm) of macroporous hydrogels prepared with 230 nm size templates. b) Composition of different macroporous hydrogels. (GHP_1_: GelMA‐HAMA‐PEGDA macroporous hydrogel with 1 wt% PEGDA concentration; GHP_1_‐int‐BP: the macroporous hydrogel with 1 wt% PEGDA concentration and Ac‐BPs modification at the inner pore surface; GHP_1_‐co‐BP: the macroporous hydrogel prepared with unmodified sacrificial templates, which functional molecules were mixed with gel matrix with 1 wt% PEGDA.) c) Schematic diagram, SEM, merge images of energy‐dispersive spectrometer (EDS), and the distribution of N, O, and P elements of macroporous hydrogels (scale: 100 μm). d) Peak of P2_P_ of P element through X‐ray photoelectron spectroscopy (XPS). Adapted with permission.^[^ ^153^ ^]^ Copyright, 2024, Wiley‐VCH GmbH.

**Figure 17:**
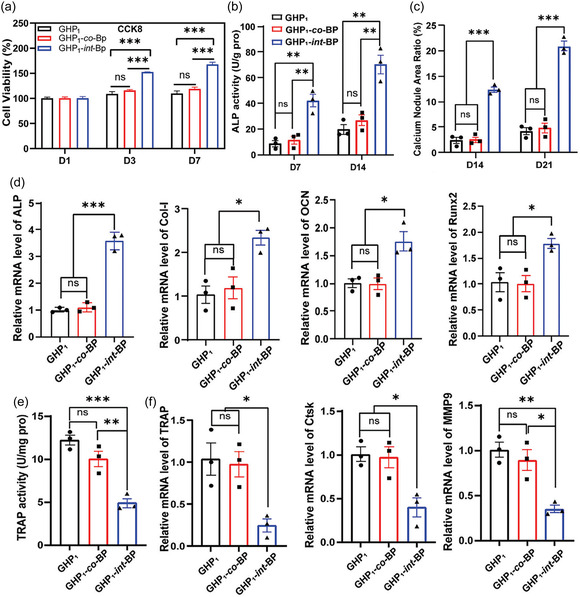
a) BMSCs viability on different macroporous hydrogels tested by the CCK‐8 assay. b) ALP activities of BMSCs cultured on different macroporous hydrogels on day 7 and day 14. c) The area of calcium nodules of BMSCs cultured on the different hydrogels on day 14 and day 21 according to Alizarin red staining. d) mRNA levels of the osteogenic genes ALP, Col‐I, OCN, and Runx2 of BMSCs cultured on the different hydrogels on day 7. e) TRAP activities of RAW264.7 cells under RANKL stimulation for osteoclast differentiation on day 5. f) Expression of the osteoclast genes TRAP, cathepsin K (Ctsk), and matrix metalloproteinase‐9 (MMP9) of RAW264.7 cells cultured on the different hydrogels on day 5. Adapted with permission.^[^ ^153^ ^]^ Copyright, 2024, Wiley‐VCH GmbH.

This strategy therefore allowed to precisely regulate the pore size and interconnected porous structure of the hydrogels (as small pores hinder nutrient transport while large pores may cause cell leakage) while successfully introducing the bioactive BPs into their internal porous surfaces (**Figure** **16**). The optimized microstructure significantly enhances stem cell migration, proliferation, and osteogenic differentiation while effectively suppressing osteoclast formation (**Figure** **17**), ultimately providing a precise and efficient artificial microenvironment for in situ bone tissue regeneration, and opening new avenues for developing functionally stable and highly bioactive bone repair materials.

Based on the above examples, MIPs hold great promise in bone tissue engineering but still face key challenges alongside vast development opportunities.

### 4.3 Challenges and Clinical Translation Osteogenic Therapy

This section synthesizes the translational hurdles associated with the two major classes of MIP‐based osteogenic technologies discussed in Sections 4.2.1 and 4.2.2.

Successful clinical translation of cell‐imprinted substrates requires stringent control of imprint fidelity and batch reproducibility, yet small deviations in template‐cell morphology, fixation chemistry, or curing conditions can alter nano/micro topographies and compromise osteoinductive cues.^[^ ^150^ ^]^ Microfluidic alignment improves geometric uniformity but cannot eliminate donor‐to‐donor variation, as mesenchymal stem cells differ in mechanosensitivity and epigenetic state.^[^ ^154^ ^]^

At the materials level, current cell‐imprinted matrices typically rely on PDMS, a polymer whose favorable cytocompatibility is offset by several bone‐specific liabilities. Its elastic modulus (tens of kPa to a few MPa) is orders of magnitude lower than cortical bone (≈10–20 GPa), rendering long‐term load‐bearing implausible.^[^ ^155^, ^156^ ^]^ PDMS is also hydrophobic, nondegradable, and poorly osteoconductive: it neither supports the hydration‐mediated degradation–regeneration synergy of native matrix nor nucleates hydroxyapatite for tight bone bonding, and chronic implantation risks fibrous encapsulation or material migration.^[^ ^157^, ^158^ ^]^ These limitations may be addressed by integrating PDMS with mechanically supportive scaffolds or applying osteoconductive surface modifications. For example, blending with calcium phosphate silicate (CPS) to create a slowly degradable, TGFβ/BMP‐activating scaffold, or coating with reduced graphene oxide (RGO) to enhance cell adhesion and support osteogenic differentiation of human adipose stem cells.^[^ ^155^, ^158^ ^]^ However, studies specifically evaluating the osteoinductive potential of such hybrid PDMS‐based cell‐imprinted constructs remain lacking, leaving a critical gap in their translational development for bone regeneration.

Finally, regulatory and ethical hurdles arise when primary human osteoblasts or patient‐derived cells serve as templates, yet current device guidelines offer no clear standards for “cellular microstructure” as an active implant function, leaving the approval pathway uncertain.

Environment‐responsive drug release is the primary translational challenge for drug‐loaded MIPs scaffolds. Bone defect sites exhibit pronounced spatiotemporal gradients throughout the infection‐to‐repair process: in the early stage, local pH decreases, ROS and protease activity increase, and then gradually return to physiological levels, while complete bone healing often takes weeks to months.^[^ ^159^ ^]^ An ideal scaffold should rapidly release antimicrobial or anti‐inflammatory drugs during the inflammatory peak, followed by sustained, low‐dose delivery of osteoinductive factors or small‐molecule agonists over a prolonged period. To achieve this, high‐affinity imprinted sites can be arranged in the outer layer, with pH/ROS/enzyme‐cleavable crosslinkers introduced to enable preferential drug release in acidic or oxidative stress environments; low‐affinity sites can be embedded in the inner core to delay later‐stage release via hydrophobic or electrostatic barriers.^[^ ^160^ ^]^ However, hierarchical imprinting strategies tailored for sequential drug release in MIP‐based delivery systems remain largely unexplored, with most current applications focused on separation technologies rather than biomedical use.^[^ ^161^, ^162^, ^163^ ^]^ Additionally, the lack of long‐term in vivo data further limits their clinical reliability and translational feasibility.

Bone regeneration relies not only on biochemical signals but also requires the scaffold to provide sufficient early mechanical support to preserve defect space, transmit mechanical load, and promote the activation of mechanosensitive pathways in osteogenic cells.^[^ ^164^ ^]^ Therefore, the co‐optimization of matrix mechanics and drug loading capacity has become the second major bottleneck. Different matrix materials offer complementary advantages, with core–shell structured MIPs scaffolds being particularly representative. For example, bioglass–chitosan microspheres imprinted with DOX form a rigid SiO_2_‐CaO core and a thermoresponsive NIPAm–MIPs shell: the glass core provides local compressive strength, the chitosan buffers interfacial brittleness, and the outer thermosensitive layer accelerates the release of antibacterial drugs at infection‐related temperature elevation (≥38 °C), followed by steady‐state sustained release.^[^ ^151^ ^]^ In this way, mechanical support is maintained while achieving temporally controlled drug delivery aligned with the pathological environment. In summary, clinically viable drug–MIPs scaffolds must simultaneously integrate environment‐responsive mechanisms and multiscale composite architectures: the outer layer is responsible for rapid drug release and initial mechanical support during inflammation, while the inner layer provides long‐term sustained release and structural maintenance. Only by validating the predictability of such integrated temporal–mechanical–pharmacokinetic strategies in in vivo models can MIPs scaffolds truly advance toward clinical application.

## 5 Conclusion and Outlook

MIPs represent a transformative approach in bone diagnostics and bone tissue engineering, offering unparalleled precision in detecting bone turnover biomarkers and advancing osteogenic treatments. By leveraging their biomimetic properties, MIPs facilitate the creation of highly specific binding sites for biomarkers and therapeutic agents, enabling the detection of BTMs, the controlled delivery of drugs or bioactive molecules, and the induction of stem cell differentiation toward osteogenesis. These innovations address the limitations of conventional diagnostic and therapeutic strategies, laying the foundation for personalized and adaptive treatments in bone health.

To fully unlock the clinical potential of MIPs in bone health, their evolution must go beyond traditional template–monomer design and embrace digitally guided molecular engineering. Looking forward, the development of intelligent MIPs will be increasingly driven by the integration of computational modeling, molecular docking, and machine learning.^[^ ^165^ ^]^ These tools offer a rational framework for optimizing template–monomer interactions, binding site geometries, and polymer network stability prior to synthesis.^[^ ^166^ ^]^ Techniques such as molecular dynamics (MD), density functional theory (DFT), and docking simulations enable detailed evaluation of interaction energies, spatial conformations, and the electronic compatibility between candidate monomers and target molecules.^[^ ^167^ ^]^ For instance, molecular docking allows the ranking of monomers based on binding affinity to target epitopes, while DFT can assess polymerization feasibility and electronic complementarity.^[^ ^168^, ^169^ ^]^ Simulated cavity analysis further guides the selection of porogenic solvents and crosslinkers to maintain template stability during imprinting.^[^ ^167^ ^]^ These computational strategies substantially reduce empirical trial‐and‐error, enhance formulation success rates, and support the development of highly selective, application‐specific MIPs platforms.

For example, Sehit et al. demonstrated the use of MD simulations to guide the design of epitope‐imprinted polymers for detecting human adenovirus. By selecting a surface‐exposed capsid epitope and evaluating its conformational stability and monomer interactions computationally, researchers synthesized MIPs through solid‐phase method with a dissociation constant of 6.48 × 10^−^^12^ m and a detection limit as low as 102 pfu mL^−1^, showing ≈2 fold higher binding affinity than conventional formulations.^[^ ^170^ ^]^

Cubuk et al. used sequence analysis, molecular docking, and MD simulations to investigate the interactions between the SARS‐CoV‐2 spike protein’ s receptor‐binding domain and commonly used functional monomers. Their computational screening identified the TEIYQAGST peptide as a stable and surface‐accessible epitope, and determined two monomers with the strongest binding affinities. Based on these insights, they proposed an epitope‐imprinted polymer design for the rapid and specific detection of SARS‐CoV‐2, highlighting the superiority of computer simulation in molecular targets identification and monomers selection.^[^ ^171^ ^]^

In another example, Liu et al. developed a pH‐responsive MIP‐based DDS for the controlled and sustained release of capecitabine (CAPE), an oral chemotherapeutic agent. By functionalizing a silica (SiO_2_) nanocarrier with 4‐formylphenylboronic acid, the system leveraged dynamic borate ester bonds between boronic acid groups and *cis*‐diols to enable acid‐triggered drug release in tumor‐like environments. The SiO_2_ substrate was further modified with fluorescein isothiocyanate for intracellular tracking. DFT calculations were used to elucidate the interaction mechanisms between CAPE and the imprinted matrix, guiding the selection of functional monomers and confirming the pH‐responsive release behavior. In vitro assays demonstrated good biocompatibility, high encapsulation efficiency, and significant cytotoxicity against cancer cells. This study highlights how theoretical modeling and rational MIPs design can enable the construction of intelligent, tumor‐microenvironment‐responsive drug carriers with translational potential.^[^ ^172^ ^]^ While these studies exemplify how computational tools can optimize drug delivery performance, the future of MIPs in regenerative medicine may extend even further, toward gene‐level regulation of bone remodeling processes.

In recent years, the application scope of MIPs has gradually expanded to include advanced biomedical fields such as gene delivery and genome editing. MIPs are now considered promising carriers for nucleic acid therapeutics, including plasmid DNA, siRNA, mRNA, and gene‐editing complexes. CRISPR/Cas9 is a transformative gene‐editing tool composed of a guide RNA (gRNA) and the Cas9 endonuclease, which work together to introduce double‐strand breaks at specific DNA loci.^[^ ^173^ ^]^ These breaks are then repaired by cellular mechanisms, enabling precise gene knockout, insertion, or modulation. In the context of bone regeneration, CRISPR technology has demonstrated efficacy in activating osteogenic transcription factors such as RUNX2, OSX, and BMP‐2 and in suppressing osteoclast‐related genes such as RANK and NFATc1 to reduce bone resorption.^[^ ^174^, ^175^, ^176^, ^177^ ^]^ Furthermore, CRISPR has also been investigated as a strategy to correct monogenic bone disorders such as osteogenesis imperfecta.^[^ ^178^ ^]^

Although current research on MIP‐mediated gene delivery remains limited, advances in polymer carrier systems provide a promising foundation upon which MIPs can build more targeted and responsive platforms. Recent studies have explored the use of hyperbranched polymer (HP) systems to address the limitations of plasmid DNA (pDNA) delivery for CRISPR/Cas9 gene editing. One such study developed a series of HP variants and demonstrated that these nonviral carriers exhibit low cytotoxicity and efficient pDNA condensation.^[^ ^179^, ^180^ ^]^

Against this backdrop, MIPs present an exciting application prospect as gene‐editing‐assisted platforms for bone repair, even though direct research in this area is currently lacking. MIPs can form highly specific imprinted cavities capable of recognizing and stabilizing key CRISPR components, such as Cas9 proteins, mRNA, or ribonucleoprotein complexes, while simultaneously protecting them from degradation and off‐target interactions. By incorporating stimuli‐responsive linkers or degradation‐sensitive structural motifs, MIP‐based carriers can achieve synchronized release of gene payloads during the local bone healing process. Moreover, MIPs can be integrated into bone repair scaffolds using advanced fabrication techniques such as 3D printing or injectable hydrogel systems. These hybrid constructs can be designed with spatial stratification, where the outer MIPs layer delivers gene silencers (e.g., CRISPRi targeting inflammatory factors), while the inner core enables long‐term release of osteogenic gene activators (e.g., CRISPRa constructs for BMP‐2 or OSX).

In summary, MIPs have progressed from simple molecular recognition elements to multifunctional, programmable platforms with broad applications in diagnostics, drug delivery, cell modulation, and bone repair scaffolds. Their intrinsic selectivity, chemical stability, and responsiveness to environmental stimuli make them key enablers of next‐generation, precision‐engineered therapeutics. However, fully realizing their clinical potential will require not only continued innovation in imprinting techniques but also rigorous in vivo validation and seamless integration with enabling technologies such as microfluidic platforms, computer‐aided modeling and gene‐editing systems. Looking ahead, interdisciplinary collaboration across polymer chemistry, materials science, computational biology, and regenerative medicine will be critical to advancing MIPs from intelligent biomaterials to clinically viable, patient‐specific solutions for bone regeneration.

## Conflict of Interests

The authors declare no conflict of interest.

## Author Contributions

**Ren Yang**: writing—original draft; methodology; data curation; formal analysis; visualization. **Xiaohan Ma**: writing—original draft; methodology; data curation; formal analysis; visualization; writing—review and editing; conceptualization; supervision; project administration; funding acquisition. **Mingcheng Xuan**: writing—original draft; methodology; data curation; formal analysis; visualization. **Yingqi Ma**: writing—original draft; methodology; data curation; formal analysis; visualization. **Jiexian Ding**: writing—original draft; methodology; data curation; formal analysis; visualization. **David Y. S. Chau**: writing—review and editing; conceptualization; supervision; project administration; funding acquisition. **Jonathan C. Knowles**: writing—review and editing; conceptualization; supervision; project administration; funding acquisition. **Feng Peng**: writing—review and editing; conceptualization; supervision; project administration; funding acquisition. **Alessandro Poma**: writing—review and editing; conceptualization; supervision; project administration; funding acquisition. All authors contributed to the article and approved the final manuscript.

## References

[1] R. Florencio‐Silva , G. R. D. S. Sasso , E. Sasso‐Cerri , M. J. Simões , P. S. Cerri , BioMed Res. Int. 2015, 2015, 421746. 26247020 10.1155/2015/421746 PMC4515490

[2] A. M. Parfitt , The Coupling of Bone Formation to Bone Resorption: A Critical Analysis of the Concept and of its Relevance to the Pathogenesis of Osteoporosis, Elsevier, Amsterdam, New York 1982, pp. 1–6. 10.1016/0221-8747(82)90002-9 7121250

[3] G. D. Roodman , Am. Cancer Soc. 1997, 80, 1557. 10.1002/(sici)1097-0142(19971015)80:8+<1557::aid-cncr5>3.3.co;2-k 9362422

[4] K. Hankenson , G. Zimmerman , R. Marcucio , Injury 2014, 45, S8. 24857030 10.1016/j.injury.2014.04.003 PMC4406220

[5] S. H. Ralston , Bone 2008, 43, 819. 18672105 10.1016/j.bone.2008.06.015

[6] B. Kim , Y. J. Cho , W. Lim , Exp. Ther. Med. 2021, 22, 1. 10.3892/etm.2021.10815 PMC8506919 34650627

[7] A. S. K. Sølling , T. Harsløf , B. Langdahl , Drugs Aging 2019, 36, 625. 31066015 10.1007/s40266-019-00675-8

[8] M. Di Bartolomeo , F. Cavani , A. Pellacani , A. Grande , R. Salvatori , L. Chiarini , R. Nocini , A. Anesi , Biology 2022, 11, 402. 35336776 10.3390/biology11030402 PMC8945722

[9] A. Naik , A. A. Kale , J. M. Rajwade , Biomater. Adv. 2024, 214008. 39213957 10.1016/j.bioadv.2024.214008

[10] Z. Du , X. Yan , Y. Liu , Y. Pei , J. Zhou , L. Zhang , D. Han , L. Chen , Exp. Gerontol. 2024, 198, 112642. 39603369 10.1016/j.exger.2024.112642

[11] J. O. Smith , A. Aarvold , E. R. Tayton , D. G. Dunlop , R. O. Oreffo , Tissue Eng. Part B 2011, 17, 307. 10.1089/ten.TEB.2011.0143 21615329

[12] K. Ramaraj , G. Amiya , P. R. Murugan , V. Govindaraj , M. Vasudevan , A. Thiyagarajan , in 2022 4th Inter. Conf. on Smart Systems and Inventive Technology (ICSSIT), IEEE, Piscataway, NJ 2022, pp. 326–333.

[13] A. F. Bonatti , C. De Maria , G. Vozzi , Polymers 2021, 13, 548. 33673361 10.3390/polym13040548 PMC7918123

[14] B. Tse Sum Bui , A. Mier , K. Haupt , Small 2023, 19, 2206453. 10.1002/smll.202206453 36650929

[15] N. Afsarimanesh , S. C. Mukhopadhyay , M. Kruger , IEEE Trans. Biomed. Eng. 2017, 65, 1264. 28858783 10.1109/TBME.2017.2744667

[16] C. Wang , A. Javadi , M. Ghaffari , S. Gong , Biomaterials 2010, 31, 4944. 20346500 10.1016/j.biomaterials.2010.02.073

[17] K. Haupt , P. X. Medina Rangel , B. T. S. Bui , Chem. Rev. 2020, 120, 9554. 32786424 10.1021/acs.chemrev.0c00428

[18] A. Poma , A. P. Turner , S. A. Piletsky , Trends Biotechnol. 2010, 28, 629. 20880600 10.1016/j.tibtech.2010.08.006

[19] J. Mahony , K. Nolan , M. Smyth , B. Mizaikoff , Anal. Chim. Acta 2005, 534, 31.

[20] H. Chen , J. Guo , Y. Wang , W. Dong , Y. Zhao , L. Sun , Adv. Sci. 2022, 9, 2202038. 10.1002/advs.202202038 PMC9534966 35908804

[21] Y. Liu , L. Wang , H. Li , L. Zhao , Y. Ma , Y. Zhang , J. Liu , Y. Wei , Prog. Polym. Sci. 2024, 150, 101790.

[22] L. Chen , X. Wang , W. Lu , X. Wu , J. Li , Chem. Soc. Rev. 2016, 45, 2137. 26936282 10.1039/c6cs00061d

[23] A. Poma , M. Whitcombe , S. Piletsky , Plastic Antibodies, Designing Receptors for the Next Generation of Biosensors, Springer, Berlin, Heidelberg 2013, pp. 105–129.

[24] G. Vasapollo , R. D. Sole , L. Mergola , M. R. Lazzoi , A. Scardino , S. Scorrano , G. Mele , Int. J. Mol. Sci. 2011, 12, 5908. 22016636 10.3390/ijms12095908 PMC3189760

[25] S. Subrahmanyam , A. Guerreiro , A. Poma , E. Moczko , E. Piletska , S. Piletsky , Eur. Polym. J. 2013, 49, 100.

[26] A. Lamaoui , J. J. García‐Guzmán , A. Amine , J. M. Palacios‐Santander , L. Cubillana‐Aguilera , Molecularly Imprinted Polymer Composites, Elsevier, Amsterdam, New York 2021, pp. 49–91.

[27] D. Hong , C. Wang , L. Gao , C. Nie , Molecules 2024, 29, 3555. 39124961 10.3390/molecules29153555 PMC11314232

[28] A. G. Ayankojo , J. Reut , V. Syritski , E. Sehit , M. Sharifuzzaman , Z. Altintas , Molecularly Imprinted Polymers: Computational Studies to Advanced Applications, Springer, Berlin, New York 2024, pp. 75–128.

[29] J. Yan , J. Huang , S. Peng , D. Sun , W. Lu , Z. Song , J. Ma , J. You , H. Fan , L. Chen , J. Chromatogr. A 2025, 1754, 466016. 40349500 10.1016/j.chroma.2025.466016

[30] F. A. Sahara , M. S. Sultana , M. K. Amin , M. Shamim Al Mamun , P. K. Dhar , S. K. Dutta , ChemistryOpen 2025, 14, e202400277. 39473328 10.1002/open.202400277 PMC11808263

[31] Z. M. Karazan , M. Roushani , Molecularly Imprinted Polymers as Artificial Antibodies for the Environmental Health: A Step Towards Achieving the Sustainable Development Goals, Springer, Berlin, New York 2024, pp. 31–52.

[32] L. M. Gonçalves , Curr. Opin. Electrochem. 2021, 25, 100640.

[33] A.‐M. Gavrilă , E.‐B. Stoica , T.‐V. Iordache , A. Sârbu , Appl. Sci. 2022, 12, 3080.

[34] W. Sukjee , C. Tancharoen , P. T. Yenchitsomanus , M. P. Gleeson , C. Sangma , ChemistryOpen 2017, 6, 340. 28638764 10.1002/open.201700037 PMC5474651

[35] A. Palma‐Cando , I. Rendón‐Enríquez , M. Tausch , U. Scherf , Nanomaterials 2019, 9, 1125. 31382661 10.3390/nano9081125 PMC6723103

[36] J. Zhu , W. Wen , Z. Tian , X. Zhang , S. Wang , Talanta 2023, 260, 124613. 37146454 10.1016/j.talanta.2023.124613

[37] T. Zidarič , D. Majer , T. Maver , M. Finšgar , U. Maver , Analyst 2023, 148, 1102. 36723087 10.1039/d2an02025d

[38] S. Mu , Y. Yang , J. Phys. Chem. B 2008, 112, 11558. 18712904 10.1021/jp8051517

[39] M. Ali , M. Bacchu , M. Daizy , C. Tarafder , M. Hossain , M. Rahman , M. Khan , Anal. Chim. Acta 2020, 1121, 11. 32493584 10.1016/j.aca.2020.05.004

[40] Y. Liu , X.‐Z. Meng , X. Luo , H.‐W. Gu , X.‐L. Yin , W.‐L. Han , H.‐C. Yi , Y. Chen , Sens. Actuators B: Chem. 2024, 410, 135682.

[41] M. Garg , N. Pamme , TrAC Trends Anal. Chem. 2024, 170, 117437.

[42] J. Ribeiro , C. Pereira , A. Silva , M. G. F. Sales , Anal. Chim. Acta 2017, 981, 41. 28693728 10.1016/j.aca.2017.05.017

[43] J. Liu , Y. Wang , X. Liu , Q. Yuan , Y. Zhang , Y. Li , Talanta 2019, 199, 573. 30952300 10.1016/j.talanta.2019.03.018

[44] T. Kaufmann , B. J. Ravoo , Polym. Chem. 2010, 1, 371.

[45] S. Piletsky , F. Canfarotta , A. Poma , A. M. Bossi , S. Piletsky , Trends Biotechnol. 2020, 38, 368. 31677857 10.1016/j.tibtech.2019.10.002

[46] F. Dickert , O. Hayden , Anal. Chem. 2002, 74, 1302. 11922297 10.1021/ac010642k

[47] P. P. Abadi , J. C. Garbern , S. Behzadi , M. J. Hill , J. S. Tresback , T. Heydari , M. R. Ejtehadi , N. Ahmed , E. Copley , H. Aghaverdi , Adv. Funct. Mater. 2018, 28, 1707378.

[48] L. M. Murray , V. Nock , J. J. Evans , M. M. Alkaisi , J. Nanobiotechnol. 2014, 12, 1. 10.1186/s12951-014-0060-6 PMC4304612 25547467

[49] J. Medlock , A. A. Das , L. A. Madden , D. J. Allsup , V. N. Paunov , Chem. Soc. Rev. 2017, 46, 5110. 28660268 10.1039/c7cs00179g

[50] I. Mutreja , T. Woodfield , S. Sperling , V. Nock , J. Evans , M. Alkaisi , Biofabrication 2015, 7, 025002. 25850524 10.1088/1758-5090/7/2/025002

[51] F. Hasannejad , L. Montazeri , J. F. Mano , S. Bonakdar , BioImpacts 2023, 14, 29945. 38938752 10.34172/bi.2023.29945 PMC11199935

[52] J. Fu , Y. J. Chuah , J. Liu , S. Y. Tan , D.‐A. Wang , ACS Biomater. Sci. Eng. 2018, 4, 4321. 33418827 10.1021/acsbiomaterials.8b00993

[53] P. Kanchanawong , D. A. Calderwood , Nat. Rev. Mol. Cell Biol. 2023, 24, 142. 36168065 10.1038/s41580-022-00531-5 PMC9892292

[54] Y. J. Chuah , Y. Zhang , Y. Wu , N. V. Menon , G. H. Goh , A. C. Lee , V. Chan , Y. Zhang , Y. Kang , Acta Biomater. 2015, 23, 52. 26026305 10.1016/j.actbio.2015.05.023

[55] A. Shakeri , S. Khan , T. F. Didar , Lab Chip 2021, 21, 3053. 34286800 10.1039/d1lc00288k

[56] S. Cai , C. Wu , W. Yang , W. Liang , H. Yu , L. Liu , Nanotechnol. Rev. 2020, 9, 971.

[57] Y. Saylan , Ö. Altıntaş , A. Denizli , Results Opt. 2023, 13, 100541.

[58] R. Karunakaran , C. L. Onorati , K. Amreen , S. Goel , P. A. Lieberzeit , Analysis Sensing 2024, 5, e202400029.

[59] J. Liu , Y. Zhang , M. Jiang , L. Tian , S. Sun , N. Zhao , F. Zhao , Y. Li , Biosens. Bioelectron. 2017, 91, 714. 28126661 10.1016/j.bios.2017.01.037

[60] Y. Saylan , A. Denizli , Micromachines 2019, 10, 766. 31717964 10.3390/mi10110766 PMC6915378

[61] S. Wagner , J. Bell , M. Biyikal , K. Gawlitza , K. Rurack , Biosens. Bioelectron. 2018, 99, 244. 28772227 10.1016/j.bios.2017.07.053

[62] M. Babaei , S. Bonakdar , B. Nasernejad , Sci. Rep. 2022, 12, 12837. 35896682 10.1038/s41598-022-17252-6 PMC9329428

[63] W. Chen , Y. Ma , J. Pan , Z. Meng , G. Pan , B. Sellergren , Polymers 2015, 7, 1689.

[64] Z. Li , J. Deng , P. Ma , H. Bai , Y. Jin , Y. Zhang , A. Dong , M. Burenjargal , J. Sep. Sci. 2024, 47, e202400441. 39385447 10.1002/jssc.202400441

[65] M. E. Nash , X. Fan , W. M. Carroll , A. V. Gorelov , F. P. Barry , G. Shaw , Y. A. Rochev , Stem Cell Rev. Rep. 2013, 9, 148. 23354660 10.1007/s12015-013-9428-5

[66] N. Minoura , K. Idei , A. Rachkov , Y.‐W. Choi , M. Ogiso , K. Matsuda , Macromolecules 2004, 37, 9571.

[67] L. Carballido , T. Karbowiak , P. Cayot , M. Gerometta , N. Sok , E. Bou‐Maroun , Chem 2022, 8, 2330.

[68] A. Poma , H. Brahmbhatt , H. M. Pendergraff , J. K. Watts , N. W. Turner , Adv. Mater. 2014, 27, 750. 25413444 10.1002/adma.201404235

[69] X. Ma , Y. Tian , R. Yang , H. Wang , L. W. Allahou , J. Chang , G. Williams , J. C. Knowles , A. Poma , J. Nanobiotechnol. 2024, 22, 715. 10.1186/s12951-024-02901-x PMC11566612 39548502

[70] T. Zhang , M. Berghaus , Y. Li , Q. Song , M. M. Stollenwerk , J. Persson , K. J. Shea , B. Sellergren , Y. Lv , Adv. Healthcare Mater. 2025, 14, 2401929. 10.1002/adhm.202401929 PMC12753134 39690809

[71] R. Liu , A. Poma , Molecules 2021, 26, 3589. 34208380 10.3390/molecules26123589 PMC8231147

[72] Z. Gu , Y. Dong , S. Xu , L. Wang , Z. Liu , Angew. Chem. 2021, 133, 2695. 10.1002/anie.202012956 PMC7898932 33078504

[73] F. Canfarotta , L. Lezina , A. Guerreiro , J. Czulak , A. Petukhov , A. Daks , K. Smolinska‐Kempisty , A. Poma , S. Piletsky , N. A. Barlev , Nano Lett. 2018, 18, 4641. 29969563 10.1021/acs.nanolett.7b03206

[74] A. E. Bodoki , B.‐C. Iacob , E. Bodoki , Polymers 2019, 11, 2085.31847103

[75] K. D. Patel , H. W. Kim , J. C. Knowles , A. Poma , Adv. Funct. Mater. 2020, 30, 2001955.

[76] G. Pan , Q. Guo , Y. Ma , H. Yang , B. Li , Angew. Chem. Int. Ed. 2013, 52. 10.1002/anie.201300733 23716433

[77] M. Caldara , G. van Wissen , T. J. Cleij , H. Diliën , B. van Grinsven , K. Eersels , J. W. Lowdon , Adv. Sens. Res. 2023, 2, 2200059.

[78] N. Leibl , K. Haupt , C. Gonzato , L. Duma , Chemosensors 2021, 9, 123.

[79] D. R. Thévenot , K. Toth , R. A. Durst , G. S. Wilson , Anal. Lett. 2001, 34, 635.

[80] P. S. Sharma , A. Pietrzyk‐Le , F. D'souza , W. Kutner , Anal. Bioanal. Chem. 2012, 402, 3177. 22302165 10.1007/s00216-011-5696-6 PMC3303047

[81] H. Yang , H. Song , Z. Suo , F. Li , Q. Jin , X. Zhu , Q. Chen , Anal. Chim. Acta 2022, 1207, 339797. 35491038 10.1016/j.aca.2022.339797

[82] E. Ozgür , Y. Saylan , S. Akgönüllü , A. Denizli , Mass‐Sensitive Based Biosensors, Biosensors, CRC Press, Boca Raton, FL 2022, pp. 89–104.

[83] S. Chunta , R. Suedee , W. Boonsriwong , P. A. Lieberzeit , Anal. Chim. Acta 2020, 1116, 27. 32389186 10.1016/j.aca.2020.04.017

[84] O. S. Wolfbeis , J. Mater. Chem. 2005, 15, 2657.

[85] J. Yan , S. Liu , D. Sun , S. Peng , Y. Ming , A. Ostovan , Z. Song , J. You , J. Li , H. Fan , Sensors 2024, 24, 7068. 39517965 10.3390/s24217068 PMC11548425

[86] Q. Song , Y. Li , L. Ma , Y. Li , Y. Lv , Adv. Healthcare Mater. 2024, 13, 2400290. 10.1002/adhm.202400290 39021323

[87] A. A. Ensafi , M. Zakery , B. Rezaei , Spectrochim. Acta Part A 2019, 206, 460. 10.1016/j.saa.2018.08.040 30172874

[88] B. van Grinsven , O. Jamieson , M. Peeters , K. Eersels , Molecularly Imprinted Polymers: Computational Studies to Advanced Applications, Springer, Berlin, New York 2024, pp. 199–220.

[89] J. McClements , L. Bar , P. Singla , F. Canfarotta , A. Thomson , J. Czulak , R. E. Johnson , R. D. Crapnell , C. E. Banks , B. Payne , ACS Sens. 2022, 7, 1122. 35416035 10.1021/acssensors.2c00100 PMC9016778

[90] P. Hill , Br. J. Orthod. 1998, 25, 101. 9668992 10.1093/ortho/25.2.101

[91] S. Iyer , D. J. Adams , Calcified Tissue Int. 2023, 113, 96. 10.1007/s00223-023-01096-x PMC10326125 37243756

[92] S. Zhu , W. Chen , A. Masson , Y.‐P. Li , Cell Discovery 2024, 10, 71. 38956429 10.1038/s41421-024-00689-6 PMC11219878

[93] Z. Wu , W. Li , K. Jiang , Z. Lin , C. Qian , M. Wu , Y. Xia , N. Li , H. Zhang , H. Xiao , MedComm 2024, 5, e657. 39049966 10.1002/mco2.657 PMC11266958

[94] P. Szulc , D. C. Bauer , R. Eastell , Marcus and Feldman's Osteoporosis, Elsevier, Amsterdam, New York 2021, pp. 1545–1588.

[95] J. W. Lee , D. Figeys , J. Vasilescu , Adv. Cancer Res. 2006, 96, 269. 10.1016/S0065-230X(06)96010-2 17161683

[96] K. Banan , B. Mostafiz , B. Safaei , S. A. Bigdeli , M. Haavisto , F. Ghorbani‐Bidkorpeh , Molecularly Imprinted Polymers: Path to Artificial Antibodies, Springer, Berlin, New York 2024, p. 163.

[97] C. Malitesta , E. Mazzotta , R. A. Picca , A. Poma , I. Chianella , S. A. Piletsky , Anal. Bioanal. Chem. 2012, 402, 1827. 21947439 10.1007/s00216-011-5405-5

[98] W. Jesadabundit , S. Jampasa , K. Patarakul , W. Siangproh , O. Chailapakul , Biosens. Bioelectron. 2021, 191, 113387. 34146970 10.1016/j.bios.2021.113387

[99] R. Radfar , E. Akin , E. Sehit , N. S. Moldovean‐Cioroianu , N. Wolff , R. Marquant , K. Haupt , L. Kienle , Z. Altintas , Anal. Bioanal. Chem. 2024, 416, 6237. 39412695 10.1007/s00216-024-05536-x PMC11541377

[100] H. Hayrapetyan , T. Tran , E. Tellez‐Corrales , C. Madiraju , ELISA: Methods and protocols, Humana, New York, NY, 2023, pp. 1–17. 10.1007/978-1-0716-2903-1_1 36795355

[101] S. Hosseini , P. Vázquez‐Villegas , M. Rito‐Palomares , S. O. Martinez‐Chapa , Enzyme‐Linked Immunosorbent Assay (ELISA) from A To Z, Springer, Singapore 2018, pp. 67–115.

[102] Z. Altintas , E. Sehit , Y. Pan , X. Ma , Z. Yang , Molecularly Imprinted Polymers: Computational Studies To Advanced Applications, Springer, Berlin, New York 2024, pp. 33–74.

[103] S.‐P. Tang , F. Canfarotta , K. Smolinska‐Kempisty , E. Piletska , A. Guerreiro , S. Piletsky , Anal. Methods 2017, 9, 2853.

[104] N. I. Wardani , T. Kangkamano , R. Wannapob , P. Kanatharana , P. Thavarungkul , W. Limbut , Talanta 2023, 254, 124137. 36463801 10.1016/j.talanta.2022.124137

[105] X. Wang , G. Chen , P. Zhang , Q. Jia , Anal. Methods 2021, 13, 1660. 33861232 10.1039/d1ay00067e

[106] M. D. Ariani , A. Zuhrotun , P. Manesiotis , A. N. Hasanah , Polym. Adv. Technol. 2024, 35, e6201. 10.1039/d4ra01018c PMC11043797 38665502

[107] Y. Wang , J. Li , L. Wang , J. Qi , L. Chen , Se pu= Chin. J. Chromatogr. 2021, 39, 134. 10.3724/SP.J.1123.2020.08008 PMC9274850 34227346

[108] Q. Ye , F. Zhou , Antifouling surfaces and materials: From Land to Marine Environment, Springer, Berlin, New York 2014, pp. 55–81.

[109] Q. Yu , Y. Zhang , H. Wang , J. Brash , H. Chen , Acta Biomater. 2011, 7, 1550. 21195214 10.1016/j.actbio.2010.12.021

[110] S. Saxena , P. Sen , L. Soleymani , T. Hoare , Adv. Sens. Res. 2024, 3, 2300170.

[111] X. Yang , Z. Liu , Y. Kong , Z.‐Z. Yin , G. Zheng , H. Zhang , Microchem. J. 2024, 201, 110613.

[112] L. Yao , C. He , S. Chen , W. Zhao , Y. Xie , S. Sun , S. Nie , C. Zhao , Langmuir 2018, 35, 1430. 30056716 10.1021/acs.langmuir.8b01621

[113] A. Savari , B. S. Tahir , A. M. Ahmed , R. Foroutan , B. Ramavandi , Results Chem. 2025, 15, 102256.

[114] M. Cui , Z. Che , Y. Gong , T. Li , W. Hu , S. Wang , Chem. Eng. J. 2022, 431, 133455.

[115] K. Ramajayam , S. Ganesan , P. Ramesh , M. Beena , T. Kokulnathan , A. Palaniappan , Biomimetics 2023, 8, 245. 37366840 10.3390/biomimetics8020245 PMC10296503

[116] Z. Liu , Q. Liu , H. Guo , J. Liang , Y. Zhang , Front. Cell Dev. Biol. 2022, 10, 837430. 35573673 10.3389/fcell.2022.837430 PMC9096102

[117] M. Beederman , J. D. Lamplot , G. Nan , J. Wang , X. Liu , L. Yin , R. Li , W. Shui , H. Zhang , S. H. Kim , J. Biomed. Sci. Eng. 2013, 6, 32. 26819651 10.4236/jbise.2013.68A1004 PMC4725591

[118] A. Oryan , A. Kamali , A. Moshiri , J. Controlled Release 2015, 215, 12. 10.1016/j.jconrel.2015.07.022 26226345

[119] K. Dwan , C. A. Phillipi , R. D. Steiner , D. Basel , Cochrane Database Syst. Rev. 2016, 10, CD005088. 27760454 10.1002/14651858.CD005088.pub4 PMC6611487

[120] L. F. de Castro , J. M. Whitlock , Z. Michel , K. Pan , J. Taylor , V. Szymczuk , B. Boyce , D. Martin , V. Kram , R. Galisteo , Bone Res. 2024, 12, 10. 38378678 10.1038/s41413-023-00311-7 PMC10879491

[121] P. Hadji , Climacteric 2012, 15, 513. 22853318 10.3109/13697137.2012.688079 PMC3793274

[122] J. Keller , P. Catala‐Lehnen , A. K. Huebner , A. Jeschke , T. Heckt , A. Lueth , M. Krause , T. Koehne , J. Albers , J. Schulze , Nat. Commun. 2014, 5, 5215. 25333900 10.1038/ncomms6215 PMC4205484

[123] J. Zhang , A. Cohen , B. Shen , L. Du , A. Tasdogan , Z. Zhao , E. J. Shane , S. J. Morrison , Proc. Natl. Acad. Sci. 2021, 118, e2026176118. 34140410 10.1073/pnas.2026176118 PMC8237660

[124] K. S. Houschyar , C. Tapking , M. R. Borrelli , D. Popp , D. Duscher , Z. N. Maan , M. P. Chelliah , J. Li , K. Harati , C. Wallner , Front. cell Dev. Biol. 2019, 6, 170. 30666305 10.3389/fcell.2018.00170 PMC6330281

[125] J. H. Kim , X. Liu , J. Wang , X. Chen , H. Zhang , S. H. Kim , J. Cui , R. Li , W. Zhang , Y. Kong , Ther. Adv. musculoskeletal Dis. 2013, 5, 13. 10.1177/1759720X12466608 PMC3582304 23514963

[126] B. Kovács , E. E. Nagy , N. N. Chendrean , B. Székely‐Szentmiklósi , Á. Gyéresi , Acta Marisiensis‐Seria Med. 2017, 63, 104.

[127] F. Yu , C. Yu , F. Li , Y. Zuo , Y. Wang , L. Yao , C. Wu , C. Wang , L. Ye , Signal Transduction Targeted Ther. 2021, 6, 307. 10.1038/s41392-021-00701-5 PMC8403677 34456337

[128] S.‐J. Seo , C. Mahapatra , R. K. Singh , J. C. Knowles , H.‐W. Kim , J. Tissue Eng. 2014, 5, 2041731414541850. 25343021 10.1177/2041731414541850 PMC4206689

[129] H. W. Kim , H. E. Kim , J. C. Knowles , Adv. Funct. Mater. 2006, 16, 1529.

[130] R. A. Perez , J.‐E. Won , J. C. Knowles , H.‐W. Kim , Adv. Drug Delivery Rev. 2013, 65, 471. 10.1016/j.addr.2012.03.009 22465488

[131] J. C. Knowles , J. Mater. Chem. 2003, 13, 2395.

[132] E. A. Abou Neel , D. M. Pickup , S. P. Valappil , R. J. Newport , J. C. Knowles , J. Mater. Chem. 2009, 19, 690.

[133] D. Laurencin , N. Almora‐Barrios , N. H. de Leeuw , C. Gervais , C. Bonhomme , F. Mauri , W. Chrzanowski , J. C. Knowles , R. J. Newport , A. Wong , Biomaterials 2011, 32, 1826. 21144581 10.1016/j.biomaterials.2010.11.017

[134] S. K. Sharma , S. Gajević , L. K. Sharma , R. Pradhan , S. Miladinović , A. Ašonja , B. Stojanović , Materials 2024, 17, 5157. 39517433 10.3390/ma17215157 PMC11546690

[135] B. Dhandayuthapani , Y. Yoshida , T. Maekawa , D. S. Kumar , Int. J. Polym. Sci. 2011, 2011, 290602.

[136] H.‐W. Kim , J. C. Knowles , H.‐E. Kim , Biomaterials 2004, 25, 1279. 14643602 10.1016/j.biomaterials.2003.07.003

[137] H. W. Kim , H. H. Lee , J. Knowles , J. Biomed. Mater. Res. Part A 2006, 79, 643. 10.1002/jbm.a.30866 16826596

[138] E. Abou Neel , I. Ahmed , J. Pratten , S. Nazhat , J. Knowles , Biomaterials 2005, 26, 2247. 15585226 10.1016/j.biomaterials.2004.07.024

[139] I. Ahmed , C. Collins , M. Lewis , I. Olsen , J. Knowles , Biomaterials 2004, 25, 3223. 14980417 10.1016/j.biomaterials.2003.10.013

[140] J. M. Muncie , V. M. Weaver , Curr. Topics Dev. Boil. 2018, 130, 1. 10.1016/bs.ctdb.2018.02.002 PMC6586474 29853174

[141] F. Gattazzo , A. Urciuolo , P. Bonaldo , Biochim. Biophys. Acta BBA: Gen. Subj. 2014, 1840, 2506. 10.1016/j.bbagen.2014.01.010 PMC4081568 24418517

[142] C. Ligorio , A. Mata , Nat. Rev. Bioeng. 2023, 1, 518. 10.1038/s44222-023-00055-3 PMC10127181 37359773

[143] A. Nazbar , S. Samani , S. Y. Kashani , A. Amanzadeh , S. Shoeibi , S. Bonakdar , J. Mater. Chem. B 2022, 10, 6816. 35775439 10.1039/d2tb00279e

[144] S. Bonakdar , M. Mahmoudi , L. Montazeri , M. Taghipoor , A. Bertsch , M. A. Shokrgozar , S. Sharifi , M. Majidi , O. Mashinchian , M. Hamrang Sekachaei , ACS Appl. Mater. Interfaces 2016, 8, 13777. 27196338 10.1021/acsami.6b03302

[145] L. B. Neves , I. S. Afonso , G. Nobrega , L. G. Barbosa , R. A. Lima , J. E. Ribeiro , Micromachines 2024, 15, 670. 38930640 10.3390/mi15060670 PMC11205751

[146] M. Babaei , B. Nasernejad , E. Sharifikolouei , M. A. Shokrgozar , S. Bonakdar , ACS Omega 2022, 7, 26353. 35936447 10.1021/acsomega.2c02206 PMC9352215

[147] K. Kamguyan , A. A. Katbab , M. Mahmoudi , E. Thormann , S. Z. Moghaddam , L. Moradi , S. Bonakdar , Biomater. Sci. 2018, 6, 189. 10.1039/c7bm00733g 29189838

[148] N. Izadi , S. Irani , S. Bonakdar , B. Ghalandari , Iran. J. Sci. Technol., Trans. A: Sci. 2022, 46, 1115.

[149] M. Pazooki , S. Bonakdar , B. Ghalandari , S. Irani , Mol. Biol. Rep. 2022, 49, 4595. 35279778 10.1007/s11033-022-07306-3

[150] S. Yazdian Kashani , M. Keshavarz Moraveji , S. Bonakdar , Sci. Rep. 2021, 11, 12130. 34108580 10.1038/s41598-021-91616-2 PMC8190060

[151] R. Khademi , M. Kharaziha , ACS Appl. Mater. Interfaces 2024, 16, 31966. 38829697 10.1021/acsami.4c03501

[152] P. Ghaffari‐Bohlouli , P. Zahedi , M. Shahrousvand , Int. J. Biol. Macromol. 2020, 165, 2363. 33091473 10.1016/j.ijbiomac.2020.10.078

[153] J. Chen , Y. Jing , Y. Liu , Y. Luo , Y. He , X. Qiu , Q. Zhang , H. Xu , Adv. Healthcare Mater. 2024, 2400897. 10.1002/adhm.202400897 38626922

[154] M. Mahmoudi , S. Bonakdar , M. A. Shokrgozar , H. Aghaverdi , R. Hartmann , A. Pick , G. Witte , W. J. Parak , ACS Nano 2013, 7, 8379. 24059979 10.1021/nn403844q

[155] J. Li , X. Liu , J. M. Crook , G. G. Wallace , Colloids Surf. B: Biointerfaces 2017, 159, 386. 28818783 10.1016/j.colsurfb.2017.07.087

[156] H. N. Kim , D.‐H. Kang , M. S. Kim , A. Jiao , D.‐H. Kim , K.‐Y. Suh , Ann. Biomed. Eng. 2012, 40, 1339. 22258887 10.1007/s10439-012-0510-y PMC5439960

[157] T. Tsuzuki , K. Baassiri , Z. Mahmoudi , A. S. Perumal , K. Rajendran , G. M. Rubies , D. V. Nicolau , Materials 2022, 15, 2313. 35329765 10.3390/ma15062313 PMC8950181

[158] T. Wu , Z. Li , Y. Chen , Q. Liu , J. Zhang , K. Yu , Y. Wang , Z. Wang , T. Gong , J. Appl. Biomater. Funct. Mater. 2021, 19, 22808000211023261. 34102914 10.1177/22808000211023261

[159] Q. Wang , Y. Gao , Y. Chen , X. Wang , Q. Pei , T. Zhang , C. Wang , J. Pan , Adv. Healthcare Mater. 2025, 14, 2404260. 10.1002/adhm.202404260 39690750

[160] L. Tang , C.‐Y. Zhao , X.‐H. Wang , R.‐S. Li , J.‐R. Yang , Y.‐P. Huang , Z.‐S. Liu , Int. J. Pharm. 2015, 496, 822. 26474963 10.1016/j.ijpharm.2015.10.031

[161] M. Yan , Y. Wu , R. Lin , F. Ma , Z. Jiang , Environ. Sci.: Nano 2021, 8, 1978.

[162] Y. Wu , H. Xia , Q. Guo , F. Ma , K. Zhang , J. Pan , Microporous Mesoporous Mater. 2021, 326, 111393. 10.1016/j.micromeso.2021.111394 PMC8400459 34483712

[163] Y. Wu , W. Xing , F. Ma , J. Gao , X. Lin , J. Lu , C. Yu , M. Yan , Chem. Eng. J. 2020, 398, 125636.

[164] Q. Ma , Z. Miri , H. J. Haugen , A. Moghanian , D. Loca , J. Tissue Eng. 2023, 14, 20417314231172573. 37251734 10.1177/20417314231172573 PMC10214107

[165] T. Karasu , F. Çalışır , S. Pişkin , E. Özgür , C. Armutcu , M. E. Çorman , L. Uzun , J. Pharm. Biomed. Anal. Open 2024, 4, 100041.

[166] N. Handayani , I. P. Cantika , Y. Setiadi , H. Rusli , A. Poma , M. A. Zulfikar , H. Rachmawati , J. Environ. Chem. Eng. 2025, 13, 117051.

[167] E. Mohsenzadeh , V. Ratautaite , E. Brazys , S. Ramanavicius , S. Zukauskas , D. Plausinaitis , A. Ramanavicius , Wiley Interdiscip. Rev.: Comput. Mol. Sci. 2024, 14, e1713.

[168] R. Boroznjak , J. Reut , A. Tretjakov , A. Lomaka , A. Öpik , V. Syritski , J. Mol. Recog. 2017, 30, e2635. 10.1002/jmr.2635 28444792

[169] T. Wungu , S. Marsha , in IOP Conf. Series: Materials Science and Engineering , IOP Publishing, Bristol, UK 2017, p. 012004.

[170] E. Sehit , G. Yao , G. Battocchio , R. Radfar , J. Trimpert , M. A. Mroginski , ACS Sens. 2024, 9, 1831. 38489767 10.1021/acssensors.3c02374 PMC11059108

[171] H. Cubuk , M. Ozbil , P. C. Hatir , Comput. Theor. Chem. 2021, 1199, 113215. 33747754 10.1016/j.comptc.2021.113215 PMC7960027

[172] Z. Guo , H. Zheng , J. Ma , G. Xu , Q. Jia , Analy. Chim. Acta 2024, 1317, 342881. 10.1016/j.aca.2024.342881 39029999

[173] M. Asmamaw , B. Zawdie , Biol.: Targets Ther. 2021, 15, 353. 10.2147/BTT.S326422 PMC8388126 34456559

[174] S.‐Y. Park , J.‐K. Lee , S.‐H. Lee , D.‐S. Kim , J.‐W. Jung , J. H. Kim , S.‐W. Baek , S. You , D.‐Y. Hwang , D. K. Han , Mater. Today Bio. 2024, 28, 101254. 10.1016/j.mtbio.2024.101254 PMC11426062 39328787

[175] G. P. Freitas , H. B. Lopes , A. T. Souza , M. P. O. Gomes , G. K. Quiles , J. Gordon , C. Tye , J. L. Stein , G. S. Stein , J. B. Lian , Gene Ther. 2021, 28, 748. 33686254 10.1038/s41434-021-00248-8 PMC8423866

[176] T. Gross , C. Jeney , D. Halm , G. Finkenzeller , G. B. Stark , R. Zengerle , P. Koltay , S. Zimmermann , Plos One 2021, 16, e0238330. 33661950 10.1371/journal.pone.0238330 PMC7932140

[177] A. S. Çakmak , S. Fuerkaiti , ACS Biomater. Sci. Eng. 2023, 9, 6175. 37796024 10.1021/acsbiomaterials.3c00506 PMC10646847

[178] V. A. Truong , M.‐N. Hsu , N. T. Kieu Nguyen , M.‐W. Lin , C.‐C. Shen , C.‐Y. Lin , Y.‐C. Hu , Nucleic Acids Res. 2019, 47, e74. 30997496 10.1093/nar/gkz267 PMC6648329

[179] K. Xiu , L. Saunders , L. Wen , J. Ruan , R. Dong , J. Song , D. Yang , J. Zhang , J. Xu , Y. E. Chen , Cells 2022, 12, 156. 36611948 10.3390/cells12010156 PMC9818138

[180] H. Lee , W.‐Y. Rho , Y.‐H. Kim , H. Chang , B.‐H. Jun , Molecules 2025, 30, 542. 39942646 10.3390/molecules30030542 PMC11820414

[181] D. Elfadil , A. Lamaoui , F. Della Pelle , A. Amine , D. Compagnone , Molecules 2021, 26, 4607. 34361757 10.3390/molecules26154607 PMC8347609

[182] B. Agnishwaran , G. Manivasagam , A. Udduttula , ACS Omega 2024, 9, 8730. 38434830 10.1021/acsomega.3c08977 PMC10905706

[183] X. Ma , J. C. Knowles , A. Poma , Pharmaceutics 2023, 15, 1140. 37242682 10.3390/pharmaceutics15051440 PMC10222044

[184] Y. Yang , X. Shen , Molecules 2022, 27.

[185] F. Deng , X.‐B. Luo , L. Ding , S.‐L. Luo , Nanomaterials for the Removal of Pollutants and Resource Reutilization, (Eds: X. Luo , F. Deng ), Elsevier, Amsterdam / New York 2019, pp. 149–178.

[186] Y. Kobayashi , Y. Nakamitsu , Y. Zheng , Y. Takashima , H. Yamaguchi , A. Harada , Polymer 2019, 177, 208.

[187] Q. Xia , Y. Yun , Q. Li , Z. Huang , Z. Liang , Des. Monomers Polym. 2017, 20, 201. 29491793 10.1080/15685551.2016.1239174 PMC5812182

[188] S. Pardeshi , S. K. Singh , RSC Adv. 2016, 6, 23525.

[189] N. Funaya , J. Haginaka , J. Chromatogr. A 2012, 1248, 18. 22695694 10.1016/j.chroma.2012.05.081

[190] G. Wulff , B. O. Chong , U. Kolb , Angew. Chem. Int. Ed. Engl. 2006, 45, 2955. 16568480 10.1002/anie.200503926

[191] G. N. Wulff , J. Liu , Acc. Chem. Res. 2012, 45, 239. 21967389 10.1021/ar200146m

[192] D. Refaat , M. G. Aggour , A. A. Farghali , R. Mahajan , J. G. Wiklander , I. A. Nicholls , S. A. Piletsky , Int. J. Mol. Sci. 2019, 20, 6304. 31847152 10.3390/ijms20246304 PMC6940816

[193] J. M. Asua , Prog. Polym. Sci. 2002, 27, 1283.

[194] M. Esfandyari‐Manesh , B. Darvishi , F. A. Ishkuh , E. Shahmoradi , A. Mohammadi , M. Javanbakht , R. Dinarvand , F. Atyabi , Mater. Sci. Eng.: C 2016, 62, 626. 10.1016/j.msec.2016.01.059 26952466

[195] O. I. Parisi , F. Francomano , M. Dattilo , F. Patitucci , S. Prete , F. Amone , F. Puoci , J. Funct. Biomater. 2022, 13, 12. 35225975 10.3390/jfb13010012 PMC8883926

[196] S. Bhogal , K. Kaur , A. K. Malik , C. Sonne , S. S. Lee , K.‐H. Kim , TrAC Trends Anal. Chem. 2020, 133, 116043.

[197] J. Zhou , N. Gan , T. Li , F. Hu , X. Li , L. Wang , L. Zheng , Biosens. Bioelectron. 2014, 54, 199. 24280050 10.1016/j.bios.2013.10.044

[198] F. Canfarotta , A. Poma , A. Guerreiro , S. Piletsky , Nat. Protoc. 2016, 11, 443. 26866789 10.1038/nprot.2016.030

[199] A. Poma , A. Guerreiro , M. J. Whitcombe , E. V. Piletska , A. P. Turner , S. A. Piletsky , Adv. Funct. Mater. 2013, 23, 2821. 26869870 10.1002/adfm.201202397 PMC4746745

[200] A. Poma , A. Guerreiro , S. Caygill , E. Moczko , S. Piletsky , RSC Adv. 2014, 4, 4203. 26722622 10.1039/C3RA46838K PMC4693954

[201] A. Gómez‐Caballero , A. Elejaga‐Jimeno , G. García del Caño , N. Unceta , A. Guerreiro , M. Saumell‐Esnaola , J. Sallés , M. A. Goicolea , R. J. Barrio , Microchim. Acta 2021, 188, 1. 10.1007/s00604-021-05029-z PMC8497319 34618242

[202] Z. Zhang , L. Ma , H. Yuan , Z. Chen , Y. Lv , Adv. Healthcare Mater. 2023, 12, 2300146. 10.1002/adhm.202300146 36737673

[203] S. Vimalraj , Gene 2020, 754, 144855. 32522695 10.1016/j.gene.2020.144855

[204] P. Magnusson , C. A. Sharp , J. R. Farley , Clin. Chim. Acta 2002, 325, 59. 12367767 10.1016/s0009-8981(02)00248-6

[205] M. Schini , T. Vilaca , F. Gossiel , S. Salam , R. Eastell , Endocr. Rev. 2023, 44, 417. 36510335 10.1210/endrev/bnac031 PMC10166271

[206] O. Zaitseva , S. Shandrenko , M. Veliky , Ukr. Biochem. J. 2015, 87, 21. 26036128 10.15407/ubj87.01.021

[207] G. Lombardi , S. Perego , L. Luzi , G. Banfi , Endocrine 2015, 48, 394. 25158976 10.1007/s12020-014-0401-0

[208] B. Şimşek , Ö. Karacaer , I. Karaca , Chin. Med. J. 2004, 117, 291. 14975218

[209] M. Saito , K. Marumo , Calcified Tissue Int. 2015, 97, 242. 10.1007/s00223-015-9985-5 25791570

[210] M. J. Seibel , S. P. Robins , J. P. Bilezikian , Trends Endocrinol. Metab. 1992, 3, 263. 18407110 10.1016/1043-2760(92)90129-o

[211] T.‐R. Kuo , C.‐H. Chen , Biomarker Res. 2017, 5, 1. 10.1186/s40364-017-0097-4 PMC5436437 28529755

[212] D. Konukoglu , Int. J. Med. Biochem. 2019, 2, 65.

[213] S. Shetty , N. Kapoor , J. D. Bondu , N. Thomas , T. V. Paul , Indian J. Endocrinol. Metabol. 2016, 20, 846. 10.4103/2230-8210.192914 PMC5105571 27867890

[214] T.‐Y. Chao , J.‐C. Yu , C.‐H. Ku , M. M. Chen , S.‐H. Lee , A. J. Janckila , L. T. Yam , Clin. Cancer Res. 2005, 11, 544. 15701839

[215] T. Takeuchi , H. Yoshida , S. Tanaka , Autoimmun. Rev. 2021, 20, 102884. 34229044 10.1016/j.autrev.2021.102884

[216] M. Wu , G. Chen , Y.‐P. Li , Bone Res. 2016, 4, 1. 10.1038/boneres.2016.9 PMC4985055 27563484

[217] B. Zhang , L. Wang , P. Song , X. Pei , H. Sun , L. Wu , C. Zhou , K. Wang , Y. Fan , X. Zhang , Mater. Des. 2021, 201, 109490.

[218] S. B. Sulaiman , T. K. Keong , C. H. Cheng , A. B. Saim , R. B. H. Idrus , Indian J. Med. Res. 2013, 137, 1093. 23852290 PMC3734714

[219] L.‐C. Gerhardt , A. R. Boccaccini , Materials 2010, 3, 3867. 28883315 10.3390/ma3073867 PMC5445790

[220] G. Fernandes , V. Abhyankar , J. M. O’Dell , J. Dent. Oral Disord. Ther. 2021, 9, 1.

[221] F. Deng , L. Liu , Z. Li , J. Liu , J. Biol. Eng. 2021, 15, 1. 33478505 10.1186/s13036-021-00255-8 PMC7818551

[222] M.‐Q. Cheng , T. Wahafu , G.‐F. Jiang , W. Liu , Y.‐Q. Qiao , X.‐C. Peng , T. Cheng , X.‐L. Zhang , G. He , X.‐Y. Liu , Sci. Rep. 2016, 6, 24134. 27071777 10.1038/srep24134 PMC4829853

[223] Y. Li , Y. Liu , R. Li , H. Bai , Z. Zhu , L. Zhu , C. Zhu , Z. Che , H. Liu , J. Wang , Mater. Des. 2021, 210, 110049.

[224] D. Zhao , T. Zhu , J. Li , L. Cui , Z. Zhang , X. Zhuang , J. Ding , Bioactive Mater. 2021, 6, 346. 10.1016/j.bioactmat.2020.08.016 PMC7475521 32954053

[225] J. H. Choi , D. K. Kim , J. E. Song , J. M. Oliveira , R. L. Reis , G. Khang , Novel Biomaterials for Regenerative Medicine, Springer Singapore, 2018, pp. 371–387.

[226] S. K. L. Levengood , M. Zhang , J. Mater. Chem. B 2014, 2, 3161. 24999429 10.1039/C4TB00027G PMC4078888

[227] Z. Li , H. R. Ramay , K. D. Hauch , D. Xiao , M. Zhang , Biomaterials 2005, 26, 3919. 15626439 10.1016/j.biomaterials.2004.09.062

[228] D. Zhang , X. Wu , J. Chen , K. Lin , Bioactive Mater. 2018, 3, 129. 10.1016/j.bioactmat.2017.08.004 PMC5935759 29744450

[229] J. Babilotte , B. Martin , V. Guduric , R. Bareille , R. Agniel , S. Roques , V. Héroguez , M. Dussauze , M. Gaudon , D. Le Nihouannen , Mater. Sci. Eng.: C 2021, 118, 111334. 10.1016/j.msec.2020.111334 33254966

[230] H.‐W. Kim , J. C. Knowles , H.‐E. Kim , J. Mater. Sci.: Mater. med. 2005, 16, 189.15744609

[231] H. W. Kim , J. C. Knowles , H. E. Kim , J. Biomed. Mater. Res. Part A 2005, 72, 136. 10.1002/jbm.a.30168 15549783

